# Oxygen-evolving photosystem II structures during S_1_–S_2_–S_3_ transitions

**DOI:** 10.1038/s41586-023-06987-5

**Published:** 2024-01-31

**Authors:** Hongjie Li, Yoshiki Nakajima, Eriko Nango, Shigeki Owada, Daichi Yamada, Kana Hashimoto, Fangjia Luo, Rie Tanaka, Fusamichi Akita, Koji Kato, Jungmin Kang, Yasunori Saitoh, Shunpei Kishi, Huaxin Yu, Naoki Matsubara, Hajime Fujii, Michihiro Sugahara, Mamoru Suzuki, Tetsuya Masuda, Tetsunari Kimura, Tran Nguyen Thao, Shinichiro Yonekura, Long-Jiang Yu, Takehiko Tosha, Kensuke Tono, Yasumasa Joti, Takaki Hatsui, Makina Yabashi, Minoru Kubo, So Iwata, Hiroshi Isobe, Kizashi Yamaguchi, Michihiro Suga, Jian-Ren Shen

**Affiliations:** 1 Research Institute for Interdisciplinary Science, Graduate School of Natural Science and Technology, Okayama University, Okayama, Japan; 2 Institute of Multidisciplinary Research for Advanced Materials, Tohoku University, Sendai, Japan; 3 RIKEN SPring-8 Center, Sayo, Japan; 4 Japan Synchrotron Radiation Research Institute, Sayo, Japan; 5 Department of Picobiology, Graduate School of Life Science, University of Hyogo, Kobe, Japan; 6 Department of Cell Biology, Graduate School of Medicine, Kyoto University, Kyoto, Japan; 7 Institute for Protein Research, Osaka University, Osaka, Japan; 8 Division of Food and Nutrition, Faculty of Agriculture, Ryukoku University, Otsu, Japan; 9 Department of Chemistry, Graduate School of Science, Kobe University, Kobe, Japan; 10 Key Laboratory of Photobiology, Institute of Botany, Chinese Academy of Sciences, Beijing, China; 11 Center for Quantum Information and Quantum Biology, Osaka University, Osaka, Japan

**Keywords:** Bioenergetics, Photosystem II

## Abstract

Photosystem II (PSII) catalyses the oxidation of water through a four-step cycle of S_*i*_ states (*i* = 0–4) at the Mn_4_CaO_5_ cluster^1–3^, during which an extra oxygen (O6) is incorporated at the S_3_ state to form a possible dioxygen^4–7^. Structural changes of the metal cluster and its environment during the S-state transitions have been studied on the microsecond timescale. Here we use pump-probe serial femtosecond crystallography to reveal the structural dynamics of PSII from nanoseconds to milliseconds after illumination with one flash (1F) or two flashes (2F). Y_Z_, a tyrosine residue that connects the reaction centre P680 and the Mn_4_CaO_5_ cluster, showed structural changes on a nanosecond timescale, as did its surrounding amino acid residues and water molecules, reflecting the fast transfer of electrons and protons after flash illumination. Notably, one water molecule emerged in the vicinity of Glu189 of the D1 subunit of PSII (D1-E189), and was bound to the Ca^2+^ ion on a sub-microsecond timescale after 2F illumination. This water molecule disappeared later with the concomitant increase of O6, suggesting that it is the origin of O6. We also observed concerted movements of water molecules in the O1, O4 and Cl-1 channels and their surrounding amino acid residues to complete the sequence of electron transfer, proton release and substrate water delivery. These results provide crucial insights into the structural dynamics of PSII during S-state transitions as well as O–O bond formation.

## Abstract (web summary)

Serial femtosecond crystallography reveals the structural dynamics of photosystem II during the S-state transitions that produce dioxygen, providing insight into electron transfer, water insertion, proton release and O–O bond formation on sub-microsecond timescales.

## Main

**Extended Data Fig. 1:**
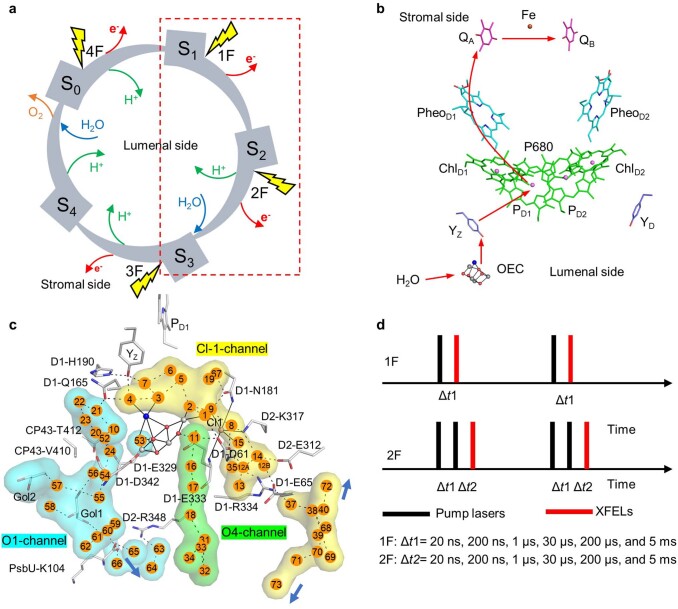
The Kok cycle and schematic of the present study. **a**, The Kok cycle. A rectangular box circled by red, dotted lines highlights the major objective of this study. **b**, Electron transfer pathway in PSII. Red arrows indicate the path of the electron. **c**, Water channels connecting the OEC with the lumenal surface. Blue arrows indicate the exits of water channels. 53′ indicates a water molecule not visible in the current model. **d**, Schematic of the pump-probe TR-SFX using one (1F) or two flashes (2F) with delay times ranging from 20 ns to 5 ms. In the 2F experiment, the interval between the first and second flash is 5 ms.

Photosystem II (PSII) produces dioxygen by extracting electrons and protons from water, which takes place at the oxygen-evolving complex (OEC), an oxo-bridged Mn_4_CaO_5_ cluster with a shape that resembles a distorted chair^2,3,8^. The Mn atoms in the OEC accumulate oxidative power through a four-step cycle of S_*i*_ states (*i* = 0–4) that is initiated by the light-driven excitation of P680, a reaction centre that is a complex of chlorophyll *a* molecules^1^ (Extended Data Fig. 1a)^1^. This is followed by a rapid charge separation that produces a pair of positive and negative charges on P680^•+^/pheophytin^•−^ (Pheo^•−^) on a picosecond timescale^9,10^. The electron is transferred from Pheo^•−^ to the primary and secondary plastoquinones Q_A_ and Q_B_ (Extended Data Fig. 1b). The P680^•+^ is then reduced by a tyrosine residue (D1-Y161; Y_Z_) located between P680 and the OEC, which is re-reduced by the OEC, pushing the OEC to a higher S_*i*_ state^11^. In conjunction with the oxidation of the OEC, protons are released in a 1:0:1:2 stoichiometry for the S_0_–S_1_, S_1_–S_2_, S_2_–S_3_ and S_3_–(S_4_)–S_0_ transitions^12–14^, and two water molecules are split to produce a dioxygen in the S_3_–(S_4_)–S_0_ transition, after which the OEC returns to its most reduced S_0_ state.

The water-splitting reaction requires a constant replenishment of water from the lumen, as well as the prompt elimination of the generated protons into the lumen. There are extensive hydrogen-bonding networks connecting the OEC with the lumen, and among these, the O1, O4 and Cl-1 channels are proposed to have essential roles in the water-splitting reaction^6,15–18^ (Extended Data Fig. 1c). (Note that the first 56 water molecules are named following a previous report^18^, and other water molecules are newly numbered; see Supplementary Table 1 for corresponding numbers in other studies). The O1 channel is a wide channel starting from a five-water cluster (W10, W20, W21, W22 and W23) that is located near O1 of the OEC (OEC-O1). This channel travels across a narrow area and ends at a giant cavity in which two glycerol molecules are found in the crystal structure^2,3^ (Extended Data Fig. 1c). The wide O1 channel might give a high mobility of water within it, and is therefore considered as a potential water inlet pathway^6,17^. By contrast, the O4 channel is a shorter channel that starts at OEC-O4 and ends at a four- or five-water cluster (Extended Data Fig. 1c). The Cl-1 channel refers to a hydrogen-bonding network mediated by Cl-1, which spans from W1 to W4, continues through D1-D61 and further extends to an ionic gate comprising D1-E65, D1-R334 and D2-E312 (Extended Data Fig. 1c). Cl^−^ ions are essential for the progression of PSII beyond the S_2_ state^19–21^, and the Cl-1 channel is thought to serve as a proton-release pathway in the S_2_–S_3_ transition^17,22,23^.

Pump-probe time-resolved femtosecond crystallography (TR-SFX) has provided a lot of information about the intermediate S-state structures of PSII (refs. ^4–7,17,24,25^). However, time-resolved structures at shorter timescales during the S_1_–S_2_ and S_2_–S_3_ transitions are lacking, and thus the sequence of OEC oxidation, proton release, electron transfer and water delivery before O6 incorporation is unclear. Here we investigate the structural dynamics during the S_1_–S_2_ and S_2_–S_3_ transitions using the pump-probe TR-SFX method at delay times (Δ*t*) of 20 ns to 5 ms (Extended Data Fig. 1d). We identify structural changes associated with electron transfer, proton release and water delivery at various regions, including Q_A_–Q_B_, Y_Z_, the OEC and the O1, O4 and Cl-1 channels. Notably, we observe the presence of a water molecule close to Ca at initial stages of the S_2_–S_3_ transition. This water molecule subsequently disappears with the concomitant increase of the O6 electron density, suggesting that it is the origin of O6. Our findings provide spatial and time-resolved snapshots of the S_1_–S_2_–S_3_ state transitions, which are important for the mechanism of O–O bond formation.

## Data quality

We obtained 14 datasets at resolutions ranging from 2.15 to 2.30 Å, with redundancy values higher than 100 even at the highest-resolution shells, after 1F or 2F (Extended Data Table 1). For all datasets, we calculated the *F*_obs_ (1F(Δ*t*1)) – *F*_obs_(Dark) and *F*_obs_ (2F(Δ*t*2)) – *F*_obs_(1F) isomorphous-difference density maps at 2.3-Å resolution. The *R*_iso_ values between the intermediate and ground states ranged from 6% to 11% (Supplementary Table 2)—sufficiently low to allow the confident detection of subtle structural changes during S_*i*_-state transitions. We observed substantial difference densities in the Q_A_–Q_B_ and OEC regions and in the proton and water channels at the electron donor side; their intensities are listed in Supplementary Table 3.

## Structural changes in the Q_A_–Fe–Q_B_ area

**Fig. 1:**
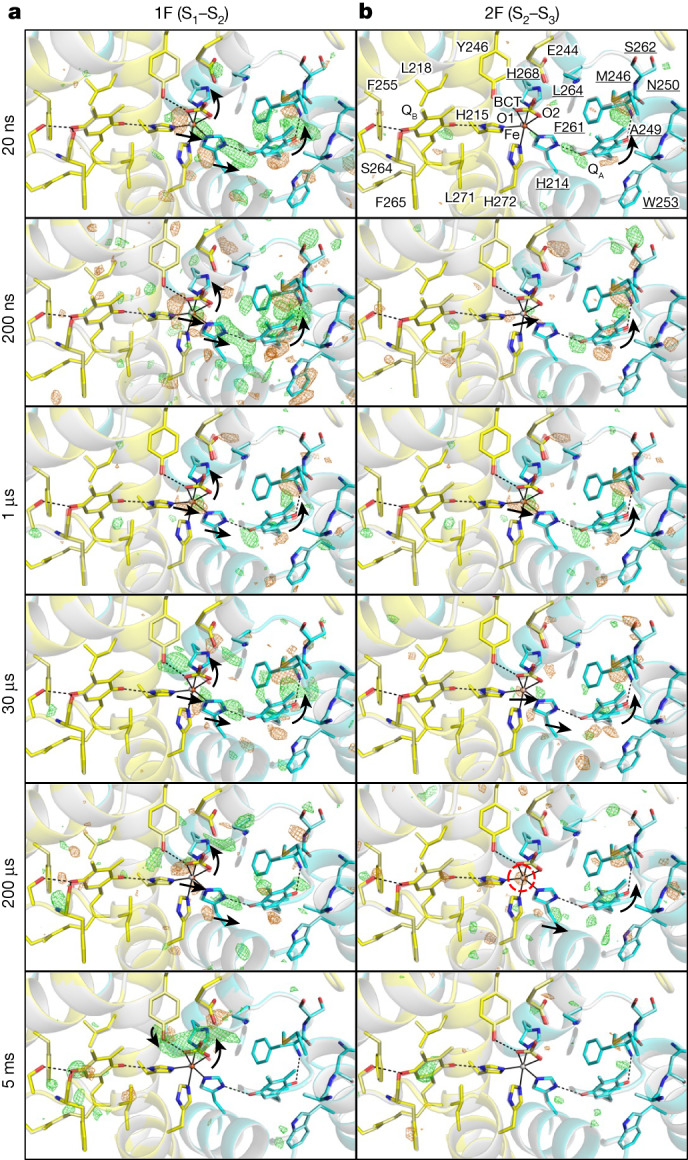
Structural dynamics in the Q_A_–Q_B_ area during S_1_–S_2_–S_3_ transitions. **a**,**b**, Structures of PSII in the Q_A_–Q_B_ area are superposed with *F*_obs_(1F) – *F*_obs_(Dark) (**a**) and *F*_obs_(2F) – *F*_obs_(1F) (**b**) difference density maps contoured at +3.5*σ* (green) and −3.5 *σ* (orange) from 20 ns to 5 ms. Ground-state models (dark in **a** and 1F in **b**) are depicted in grey, and the D1 and D2 proteins in the intermediate structures are shown in yellow and cyan, respectively. Residues of D1 and D2 are depicted without and with underlines, respectively. Hydrogen bonds are shown by black dotted lines. Black solid lines link the cofactors in PSII and their ligands. Black arrows indicate structural changes based on the refined models. The ordered and disordered atoms (non-haem iron in this figure and water molecules in the other figures) in the intermediate structures are encircled by cyan- and red-dotted lines, respectively. These nomenclature, hydrogen bonds, ligands of cofactors and black arrows are used in the other figures, unless otherwise stated.

Q_A_ and Q_B_ are linked to the non-haem iron through hydrogen bonds with D2-H214 and D1-H215, forming an iron–quinone complex. The carbonyl oxygens of the Q_A_ and Q_B_ heads are also hydrogen-bonded to D2-F261 and to D1-F265/D1-S264, respectively (Fig. 1).

Large difference densities appear on the Q_A_ side at Δ*t*1 = 20 ns and Δ*t*1 = 200 ns, become weak at Δ*t*1 = 1 µs to Δ*t*1 = 200 µs and vanish at Δ*t*1 = 5 ms (Fig. 1a and Supplementary Video 1). These changes correspond to the formation of Q_A_^−^, the oxidation of Q_A_^−^ to Q_A_ and the completion of Q_A_^−^ oxidation, respectively. The formation of Q_A_^−^ causes the counterclockwise rotation of its head group, concomitant with similar rotations or movements of D2-F261, D2-W253 and D2-H214, which surround Q_A_ (Fig. 1a and Supplementary Video 1). The formation of Q_A_^−^ also induces a shift of the non-haem iron by about 0.2 Å towards Q_A_. The pair of positive and negative difference densities around the non-haem iron is strongest at Δ*t*1 = 20 ns and Δ*t*1 = 200 ns, which is much faster than the time needed for the reduction of Fe^3+^ by Q_A_^−^ (7 µs in refs. ^26,27^), indicating that the movement of the non-haem iron is caused not by its reduction but rather by the attraction of electropositive Fe^3+^ to Q_A_^−^. The diminishing difference densities around Q_A_ and the non-haem iron at Δ*t*1 = 1 µs to Δ*t*1 = 200 µs suggest that the attraction between the non-haem iron and Q_A_^−^ is decreased and that the electron on Q_A_^−^ is transferred to the non-haem iron (Fig. 1a and Supplementary Video 1). By Δ*t*1 = 5 ms, these difference densities disappear entirely, indicating the completion of the electron transfer together with the restoration of Q_A_ and the non-haem iron.

The distances from two carbonyl oxygens of bicarbonate (BCT; BCT-O1 and BCT-O2) to the non-haem iron increase from 2.16 Å and 2.28 Å in the dark state to 2.43 Å and 2.44 Å after 5 ms of 1F (Fig. 1a, Extended Data Table 2 and Supplementary Video 1). These increases most likely reflect changes in the binding environment of BCT owing to the reduction of the non-haem iron. At Δ*t*1 = 5 ms, a large positive difference density appears between BCT and D1-Y246 (Fig. 1a and Supplementary Video 1), consistent with our previous discovery^25^, and the distance between D1-Y246 and BCT-O1 decreases from 3.21 Å to 2.82 Å. At Δ*t*1 = 200 μs to Δ*t*1 = 5 ms, the Q_B_ head shifts slightly, which might be a result of the movement of BCT or the partial reduction of Q_B_ by Q_A_^−^(ref. ^28^).

From Δ*t*2 = 20 ns to Δ*t*2 = 30 µs, the Q_A_ head rotated in a counterclockwise direction, and this was accompanied by a movement of the non-haem iron towards Q_A_—structural changes similar to those observed after 1F. However, the difference densities associated with these structural changes were much weaker after 2F (Fig. 1 and Supplementary Video 2). The non-haem iron remains Fe^2+^ at 5 ms after photoreduction by 1F, because its re-oxidation by ferricyanide takes 20 s (ref. ^27^) (Extended Data Fig. 1d). For this reason, the electron of Q_A_^−^ does not travel to the non-haem iron, but rather travels directly to Q_B_ after 2F, resulting in the absence of difference density on BCT and the appearance of positive difference density on the Q_B_ head at Δ*t*2 = 5 ms (ref. ^4^) (Fig. 1b and Supplementary Video 2). The non-haem iron becomes disordered at Δ*t*2 = 200 µs but ordered by Δ*t*2 = 5 ms, which is presumably related to electron transfer from Q_A_^−^ to Q_B_ during this time.

## Structural changes around Y_Z_

**Fig. 2:**
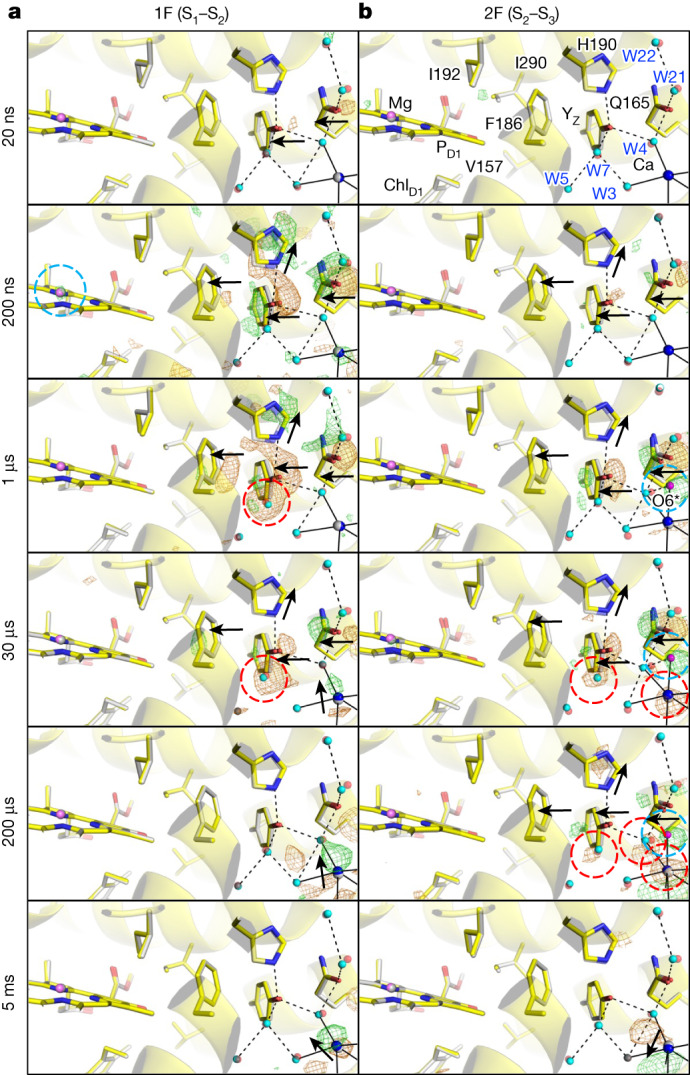
Structural dynamics in the Y_Z_ area during S_1_–S_2_–S_3_ transitions. **a**,**b**, Structures of PSII in the Y_Z_ area are superposed with *F*_obs_(1F) – *F*_obs_(Dark) (**a**) and *F*_obs_(2F) – *F*_obs_(1F) (**b**) difference density maps contoured at +4.0*σ* (green) and −4.0*σ* (orange), with delay times from 20 ns to 5 ms. Water molecules at ground and intermediate states are depicted by red and cyan spheres, respectively. The Mg atom in P_D1_ is shown as a grey sphere for the ground structure and a violet sphere for the intermediate structure. O6* and O6 (in the other figures) are depicted in magenta, and the Ca ion of the OEC is in blue. The same colour scheme is used in other figures, unless otherwise stated.

**Extended Data Fig. 2:**
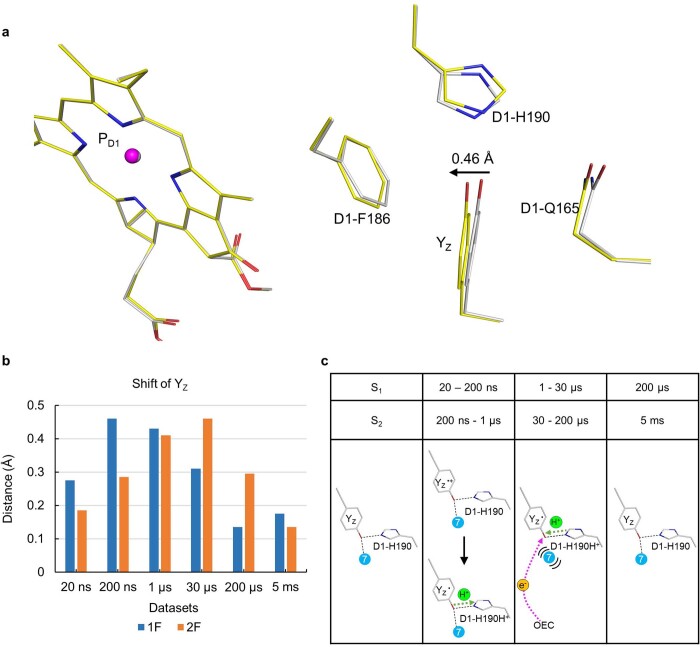
Redox status of Y_Z_. **a**, Overlap of the structures of dark (grey stick) and 1F (200 ns) (yellow stick). The black arrow indicates the direction of the Y_Z_ shift, with 0.46 Å representing the shift distance of the phenolic oxygen. **b**, Distances of the shift of the phenolic oxygen at different time points after 1F and 2F. **c**, Redox status of Y_Z_ at various time points following 1F and 2F. The black arrow at Δ*t*1 = 20–200 ns and Δ*t*2 = 200 ns–1 μs (and possibly between 1–30 μs) indicates the transformation of Y_Z_. Dotted arrows in green and magenta represent the translocations of proton and electron, respectively. The cyan sphere labelled with ‘7’ represents a water molecule W7, and curves surrounding it at Δ*t*1 = 1–30 μs and Δ*t*2 = 30–200 μs indicate its disordered structure.

D1-V157, D1-F186, D1-I192 and D1-I290 lie between Y_Z_ and P_D1_ (P680 at the D1 side) (Extended Data Figs. 1b and 2), and Y_Z_ is connected to the O1 channel through W4 and D1-Q165, to the Cl-1 channel through W7 and to the OEC through W3, W4 and W7 (Extended Data Fig. 1c). Y_Z_ forms a short (low-barrier) hydrogen bond with D1-H190 (2.44 Å in the Protein Data Bank (PDB) under accession code 3WU2; ref. ^2^), through which the phenolic proton of Y_Z_^•+^ migrates to D1-H190, forming Y_Z_^•^–D1-H190^+^ during the S_*i*_-state transitions^11,29–31^. At Δ*t*1 = 20 ns, two negative difference densities first appear adjacent to D1-Q165 and Y_Z_, and at Δ*t*1 = 200 ns, pairs of positive and negative difference densities appear over D1-Q165, Y_Z_ and D1-F186, indicating their correlated movements towards P680 (Fig. 2a and Extended Data Fig. 2a,b). These movements might be in preparation for the subsequent electron transfer from Y_Z_ to P680. Simultaneously with these movements, a positive difference density appears on the Mg atom of P_D1_ (Fig. 2a and Supplementary Table 3), which might reflect the re-reduction of P680^+^ by Y_Z_.

**Extended Data Fig. 3:**
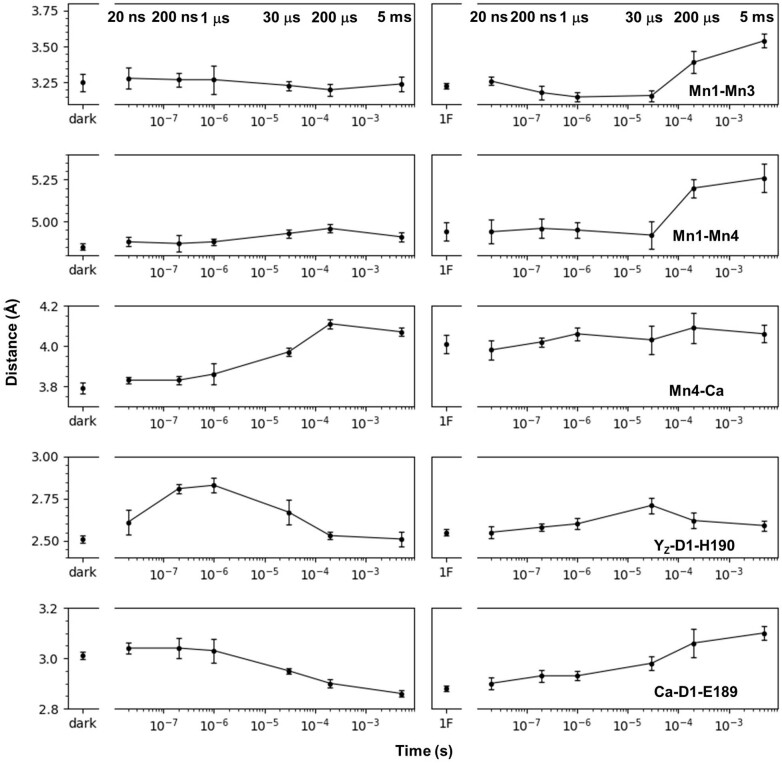
Variance in inter-atomic distances during S_1_–S_2_–S_3_ transitions. Inter-atomic distances are measured at all time points collected after 1F (left) and 2F (right). The mean values for the error bars are obtained with the structures derived from 100% indexed images. The error bars are estimated using a resampling approach, with the standard deviations determined using ten structures derived from ten sub-datasets, each comprising 75% randomly selected indexed images (see Methods for more details).

D1-H190 moves away from Y_Z_ at Δ*t*1 = 200 ns, which, together with the movement of Y_Z_ toward P680, causes the elongation of the hydrogen bond between Y_Z_ and D1-H190 from 2.51 Å to 2.80 Å at Δ*t*1 = 200 ns (Fig. 2a, Extended Data Fig. 3 and Supplementary Video 3). These changes suggest that Y_Z_ is first oxidized by P680^•+^ and subsequently deprotonated, forming a Y_Z_^•^/D1-H190^+^ species, with the time constant of Y_Z_ oxidation consistent with that reported for P680^•+^ reduction in the S_1_–S_2_ transition^31,32^. At Δ*t*1 = 1 µs and Δ*t*1 = 30 µs, difference densities on D1-Q165, Y_Z_ and D1-H190 decrease, indicating that they have moved to their original locations. In addition, a strong negative difference density appears on W7, suggesting that W7 is disordered during this period (Fig. 2a and Supplementary Video 3). By Δ*t*1 = 200 µs, all difference densities vanish at the Y_Z_ area, indicating the restoration of all residues and water, and the Y_Z_–D1-H190 distance returns to 2.53 Å (Fig. 2a, Extended Data Fig. 3, Extended Data Table 2 and Supplementary Video 3). The trajectories of the Y_Z_ area at Δ*t*1 = 1 µs and Δ*t*1 = 30 µs correspond to the re-reduction and re-protonation of Y_Z_^•^ to Y_Z_, which completes by Δ*t*1 = 200 µs, consistent with the 55–85-µs half-life of Y_Z_^•+^ re-reduction by the OEC in the S_1_–S_2_ transition^33,34^.

After 2F, difference densities start to appear only after Δ*t*2 = 200 ns (Fig. 2b and Supplementary Movie 4). These lagged difference densities likely correspond to the slower and biphasic 50-ns and 280-ns components of the P680^+^ decay in the S_2_–S_3_ transition^31,32^. This delay might arise from the reduced rate of electron transfer to P680^•+^ owing to the accumulation of a positive charge on the OEC. Difference densities on Y_Z_ and D1-Q165 increase at Δ*t*2 = 1 µs, and the Y_Z_–D1-H190 distance increases slightly from Δ*t*2 = 0 to Δ*t*2 = 1 µs (Extended Data Fig. 3, Extended Data Table 2 and Supplementary Video 4). These findings suggest the oxidation of Y_Z_ and a potential proton transfer from Y_Z_ to D1-H190 at Δ*t*2 = 1 µs, which is similar to that observed at Δ*t*1 = 20 ns–200 ns. At Δ*t*2 = 30 µs, difference densities on Y_Z_ and D1-Q165 decrease, indicating the re-reduction of Y_Z_^•^ by the OEC. The difference density on Y_Z_ becomes even weaker at Δ*t*2 = 200 µs, but is still present (Fig. 2b and Supplementary Video 4), indicating that the reduction of Y_Z_ is not yet complete, which is compatible with the half-life of 140–90 µs of Y_Z_ reduction^33,34^. Y_Z_ reduction is completed by 5 ms, and difference densities disappear at the Y_Z_ area and all residues and water molecules are restored (Fig. 2b and Supplementary Video 4). The time-resolved redox states of Y_Z_ after 1F and 2F that are described above are summarized in Extended Data Fig. 2c.

## Oxidation of the OEC during the S_1_–S_2_ transition

**Fig. 3:**
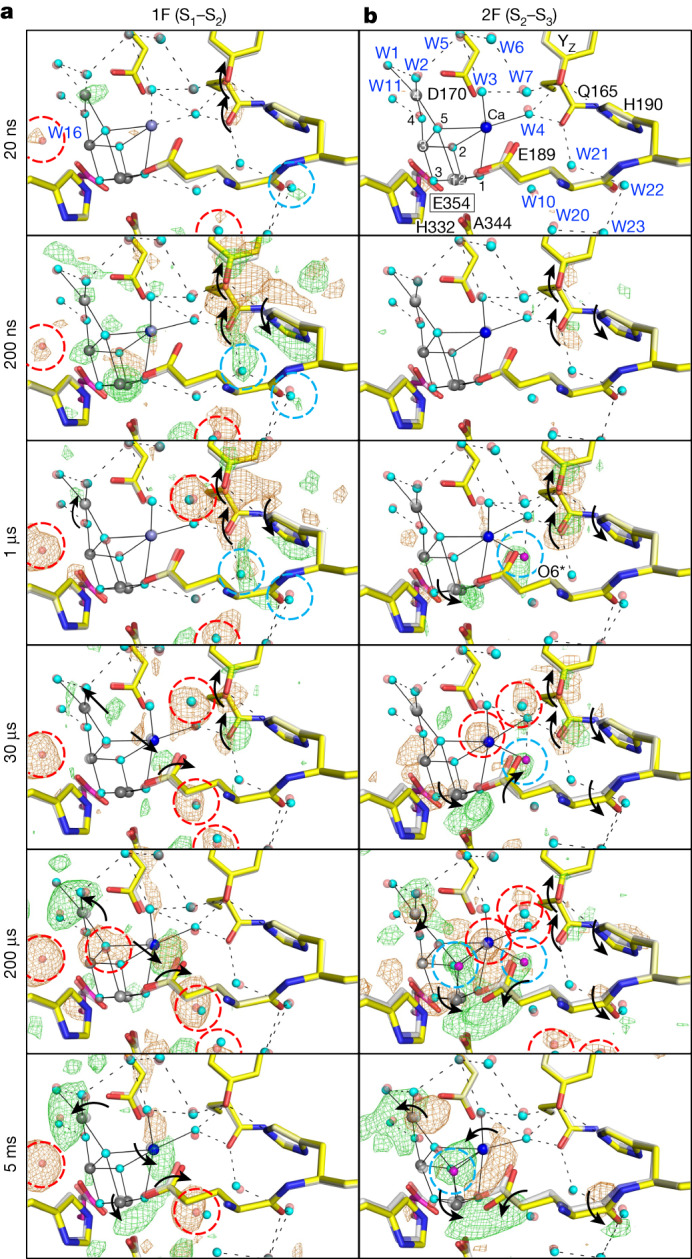
Structural dynamics of the OEC during S_1_–S_2_–S_3_ transitions. **a**,**b**, Structures of PSII in the OEC area are superposed with *F*_obs_(1F) – *F*_obs_(Dark) (**a**) and *F*_obs_(2F) – *F*_obs_(1F) (**b**) difference density maps contoured at +4.0*σ* (green) and −4.0*σ* (orange), with delay times from 20 ns to 5 ms. Residues of CP43 (a subunit of PSII) are shown in magenta and encircled by rectangles. The oxo-oxygen in the OEC and ligand waters are linked to the metal ions by black solid lines. The colour scheme used for other residues and atoms is the same as in Figs. 1 and 2.

Notable positive difference densities first appear on Mn4 and subsequently cover all four Mn and one Ca ions at Δ*t*1 = 20 ns–200 ns before OEC oxidation (Fig. 3a and Supplementary Video 3). Nevertheless, metal–metal distances remain largely unchanged (Extended Data Fig. 3 and Extended Data Table 2), suggesting a possible charge rearrangement on the OEC triggered by the electrostatic effect of the oxidized Y_Z_^•+^/Y_Z_. At Δ*t*1 = 1 µs, difference densities on Mn1–Mn3 and Ca vanish, whereas that on Mn4 continues (Fig. 3a and Supplementary Video 3). At Δ*t*1 = 30 µs, paired negative and positive difference densities appear on two sides of Ca, indicating that Mn4 and Ca move outwards from the OEC, causing an increase in the Mn4–Ca distance from 3.83 Å at Δ*t*1 = 200 ns to 3.96 Å at Δ*t*1 = 30 µs. By Δ*t*1 = 200 µs, the difference densities in the Y_Z_ area vanish completely, whereas those surrounding Mn4 and Ca increase, and the Mn4–Ca distance further extends to 4.10 Å (Fig. 3a, Extended Data Fig. 3, Extended Data Table 2 and Supplementary Video 3). The results suggest that Mn4(III) of the OEC donates one electron to Y_Z_^•^ at Δ*t*1 = 1 µs to Δ*t*1 = 200 µs. At the completion of Mn4 oxidation by Δ*t*1 = 200 µs, a negative difference density emerges on O5, suggesting its instability, which is subsequently stabilized at Δ*t*1 = 5 ms. In addition, at Δ*t*1 = 5 ms, a positive difference density appears near Mn1 but outside of the OEC, suggesting the movement of Mn1 away from the OEC. These structural changes might stabilize the positive charge on the OEC.

In correlation with the outward movement of Ca from Δ*t*1 = 30 µs to Δ*t*1 = 5 ms, one of the carboxyl oxygens of D1-E189 located close to Ca shifts slightly away from Ca. Because the movement of Ca is larger than that of D1-E189, the Ca–D1-E189 distance decreases from 3.02 Å (Δ*t*1 = 1 µs) to 2.86 Å (Δ*t*1 = 5 ms) (Fig. 3a, Extended Data Fig. 3, Extended Data Table 2 and Supplementary Video 3). In addition, W10, which is located in the proximity of D1-E189, becomes disordered in the same time range, and this correlates with the motion of Ca and D1-E189.

## Insertion of O6 in the S_2_–S_3_ transition

**Extended Data Fig. 4:**
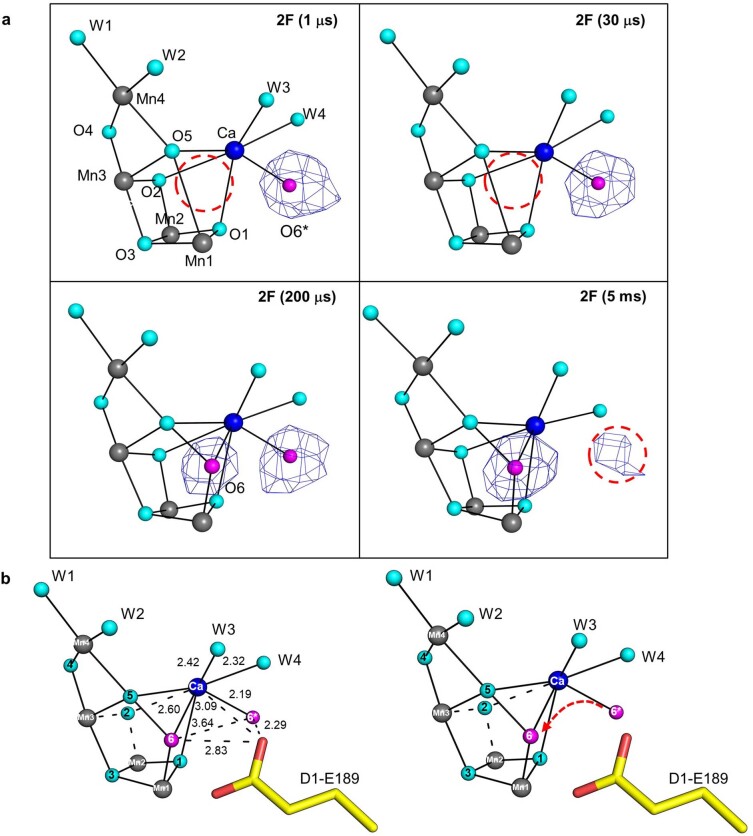
Translocation of O6* to O6. **a**, Polder omit maps contoured at +3.0*σ* (blue mesh) of O6* and O6 are superposed with OEC models after 2F. The red dashed circles indicate positions where O6 or O6* is either absent or weak during that specific time point. **b**, Translocation pathway of O6*. Numbers represent the inter-atomic distances in angstrom (Å). The red line represents the potential translocation pathway for O6*.

No difference density appears on the OEC at Δ*t*2 ≤ 200 ns, suggesting that no structural changes to the OEC occur in this time range (Fig. 3b and Supplementary Video 4). One notable positive difference density—designated as O6*—emerges approximately 2.2 Å away from Ca during Δ*t*2 = 1 µs to Δ*t*2 = 200 µs, and disappears by Δ*t*2 = 5 ms, with the concomitant increase of the O6 density from Δ*t*2 = 200 µs to Δ*t*2 = 5 ms (Fig. 3b, Extended Data Fig. 4a, Supplementary Table 3 and Supplementary Video 4). These observations suggest that O6* is the origin of O6, and that O6* binds to Ca at Δ*t*2 = 1 µs to Δ*t*2 = 30 µs, translocates to O6 at Δ*t*2 = 200 µs and completes its translocation by Δ*t*2 = 5 ms.

**Extended Data Fig. 5:**
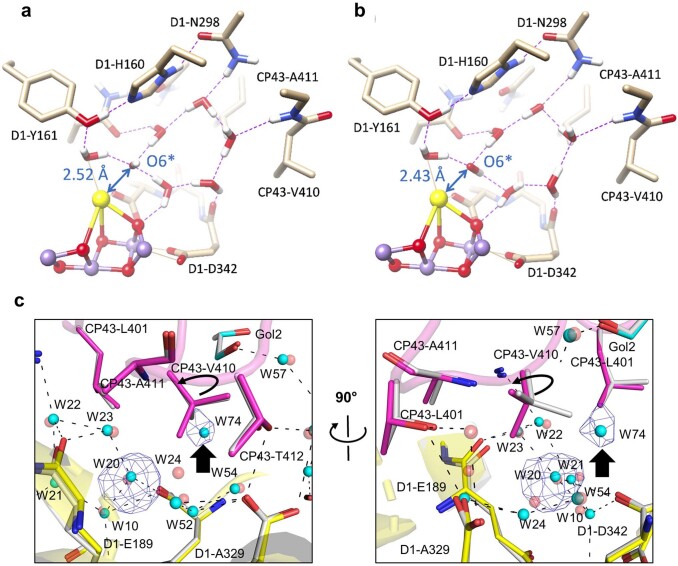
Two representative hydrogen-bonding arrangements modelled by DFT calculations, and the rotation of CP43-V410 and appearance of one new water molecule. **a**,**b**, O6* is modelled as a hydroxide anion bound to Ca^2+^ in the S_2_ state: one having a hydrogen bond between O6* and Glu189 (not depicted) (**a**), and the other one lacking this hydrogen bond (**b**). It is unstable if O6* is assumed to be a water molecule. Hydrogen atoms bonded to carbon atoms are omitted for clarity. **c**, Polder omit maps on W20 and W74 contoured at +3.5*σ* (blue mesh) superimposed with PSII structures of 1F state (grey sticks and red spheres) and 2F (5 ms) (magenta sticks for CP43 and yellow sticks for D1, as well as cyan spheres for water molecules).

At Δ*t*2 ≤ 1 µs, there are no difference densities among the neighbouring water molecules of O6*, indicating that O6* does not originate from any stable water molecules nearby. Instead, it is likely to be derived from an aqueous water—specifically, W10—located 2.5 Å away from O6* in the S_1_ state, and becomes disordered at Δ*t*1 = 30 µs–5 ms (Fig. 3 and Supplementary Video 3). The 2.2-Å distance between O6* and Ca indicates that O6* could be a hydroxide ion (OH^−^) rather than a water molecule, because water molecules W3 and W4 bind to Ca at distances ranging from 2.4 to 2.6 Å. Indeed, theoretical calculations indicate that an OH^−^ ion positioned close to O6* in the S_2_ state (Extended Data Fig. 5a,b) exhibits low energy and high stability, whereas the placement of a water molecule is not feasible. The deprotonation of water most likely occurs at Δ*t*2 = 200 ns–1 µs, during which time the simultaneous existence of Y_Z_^•+^/Y_Z_^•^ and OEC^+^ might collectively promote the deprotonation of the O6* precursor. Consequently, the resulting OH^−^ ion binds to Ca, neutralizing the positively charged OEC^+^. The achievement of a neutral OEC is crucial for the donation of an electron from the OEC to Y_Z_^•^, because it is energetically unfavourable for the OEC^+^ to continuously lose one more electron to Y_Z_ and transform into OEC^2+^. The subsequent transfer of one electron from the OEC to Y_Z_^•^, occurring at Δ*t*2 = 30 μs and Δ*t*2 = 200 μs, leads to a decrease in the difference densities at the Y_Z_ region and a simultaneous increase in the difference densities on the OEC (Fig. 3b, Supplementary Table 3 and Supplementary Video 4). The presence of paired positive and negative difference densities on both sides of Mn1 and Mn4 indicates outward movements of Mn1 and Mn4 in the OEC (Fig. 3b and Supplementary Video 4), which results in an increase in the Mn1–Mn4 distance from 4.94 Å (Δ*t*2 = 30 μs) to 5.22 Å (Δ*t*2 = 200 μs), as well as an increase in the Mn1–Mn3 distance from 3.16 Å (Δ*t*2 = 30 μs) to 3.38 A (Δ*t*2 = 200 μs) (Extended Data Fig. 3 and Extended Data Table 2). In addition, the single negative difference density on Ca suggests the disorder of Ca (Fig. 3b and Supplementary Video 4). Of note, at Δ*t*2 = 200 μs, while O6* is still present, a positive difference density emerges in the location of O6, indicating that O6 is incorporated into the OEC. The structural changes to the OEC at Δ*t*2 = 30 μs and Δ*t*2 = 200 μs can be explained as follows: Mn1 undergoes oxidation from Mn1(III) to Mn1(IV), thereby attracting the negatively charged O6*, resulting in its translocation to the O6 position and the disorder of Ca. Simultaneously, Mn1 and Mn4 move outwards to create room for O6 (the outward movement of Mn1 might also be triggered by its own oxidation). At Δ*t*2 = 200 μs, the translocation of O6* is not yet complete, resulting in simultaneous observations of both O6* and O6.

We observed no apparent difference density on W3 at all time points (Fig. 3b, Supplementary Table 3 and Supplementary Video 4), which is inconsistent with a role of W3 as the entry point for the origin of O6 (refs. ^7,35,36^). W4 also seems an unlikely candidate for an entry point owing to spatial constraints, because W4 needs to pass through W3 to reach the O6 position (Extended Data Fig. 4b). The remaining potential pathway is a direct translocation of O6* to the O6 site (Extended Data Fig. 4b). Although the 3.08-Å distance between D1-E189 and Ca might not allow the passage of O6*, this distance represents the average distance observed both in PSII molecules that have successfully completed the O6* translocation and in those that have not, but not in PSII in which O6* is being translocated. Therefore, this distance might transiently extend when O6* is passing. Furthermore, an OH^−^ ion is smaller than a water molecule, so the direct translocation of O6* (OH^−^) is possible. By Δ*t*2 = 5 ms, the translocation is completed, and O6 becomes the eighth ligand to Ca and the sixth ligand to Mn1 (Fig. 3b and Supplementary Video 4). The negative difference density near Ca indicates the inward movement of Ca towards the centre of the OEC. By contrast, Mn1 and Mn4 move further outwards from the OEC, which further opens the OEC (Fig. 3b, Extended Data Fig. 3, Extended Data Table 2 and Supplementary Video 4).

**Extended Data Fig. 6:**
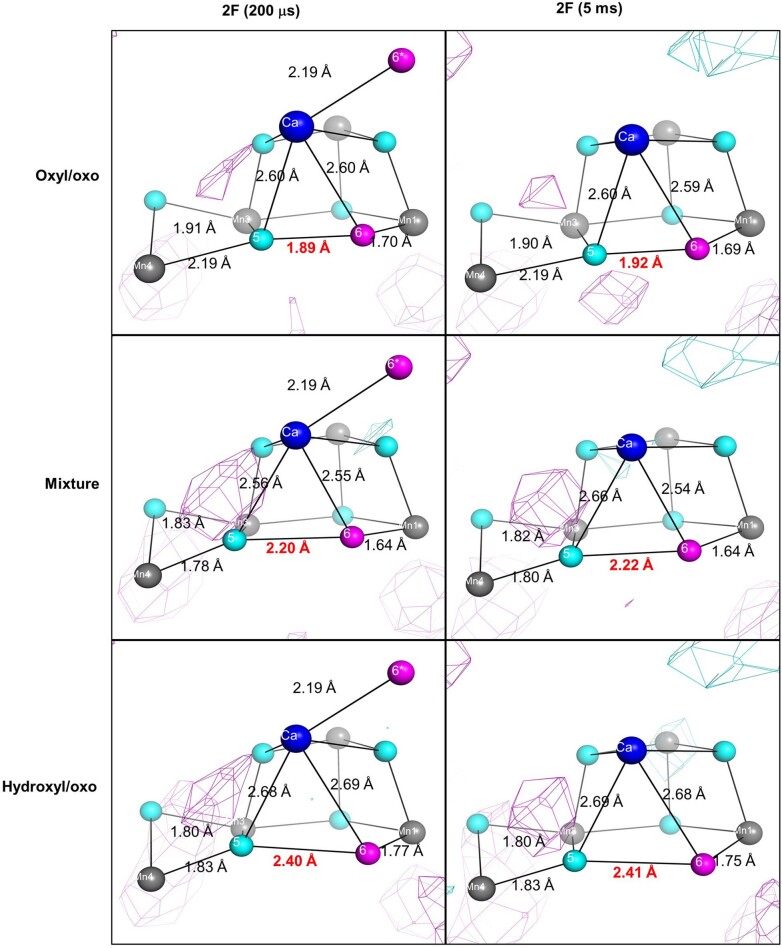
Examinations of the oxyl/oxo and hydroxyl/oxo species between O5 and O6. The m*F*_o_-D*F*_c_ maps contoured at +2.5*σ* (cyan) and −2.5*σ* (magenta) superposed with OEC structures of 2F (200 µs) (left) and 2F (5 ms) (right). O5/O6 at distances of 1.9 Å (oxyl/oxo, upper side), 2.4 Å (hydroxyl/oxo, bottom) and 2.2 Å (mixture of the two coupling species, middle) are examined. On the basis of the residual densities, the distance of 1.9 Å fits best with the electron density at 200 μs, whereas it is difficult to distinguish between the distances of 1.9 Å–2.4 Å at 5 ms.

Determining the accurate positions of O5 and O6 using electron density alone at the current 2.25-Å resolution is challenging, owing to the influence of mixed populations of different S_*i*_ states and the presence of neighbouring electron-rich metal ions. To refine the structures of OEC at Δ*t*2 = 200 μs and Δ*t*2 = 5 ms, we chose three O5–O6 distances of 1.9 Å, 2.4 Å and 2.2 Å, respectively, corresponding to oxyl/oxo, hydroxyl/oxo and deprotonated hydroxyl/oxo coupling species^37^. The optimal positions of O5 and O6 were determined with the smallest residual densities in the m*F*_o_-D*F*_c_ map, which showed an O5–O6 distance of 1.9 Å at Δ*t*2 = 200 μs, whereas the residual densities at Δ*t*2 = 5 ms were almost comparable for the O5–O6 distances of 1.9–2.4 Å (Extended Data Fig. 6). This suggests the existence of a mixed species at room temperature—different from what is observed at low temperature^6^. To maintain consistency, we set the O5–O6 distance at 1.9 Å for both Δ*t*2 = 200 μs and Δ*t*2 = 5 ms. We note that the OEC at Δ*t*2 = 5 ms is more open as compared with the structure predicted by theoretical calculations and the OEC structure solved at cryo-temperature^6,37^, as evidenced by the lengthened Mn1–Mn3 distance of 3.5 Å observed here. This might leave some room for a hydroxyl/oxo coupling mechanism, and there is a crystallographic debate regarding the existence of O6/Ox in the S_3_ structure^38^ (Supplementary Fig. 1 and Supplementary Discussion).

## Water inlet from the O1 channel

**Extended Data Fig. 7:**
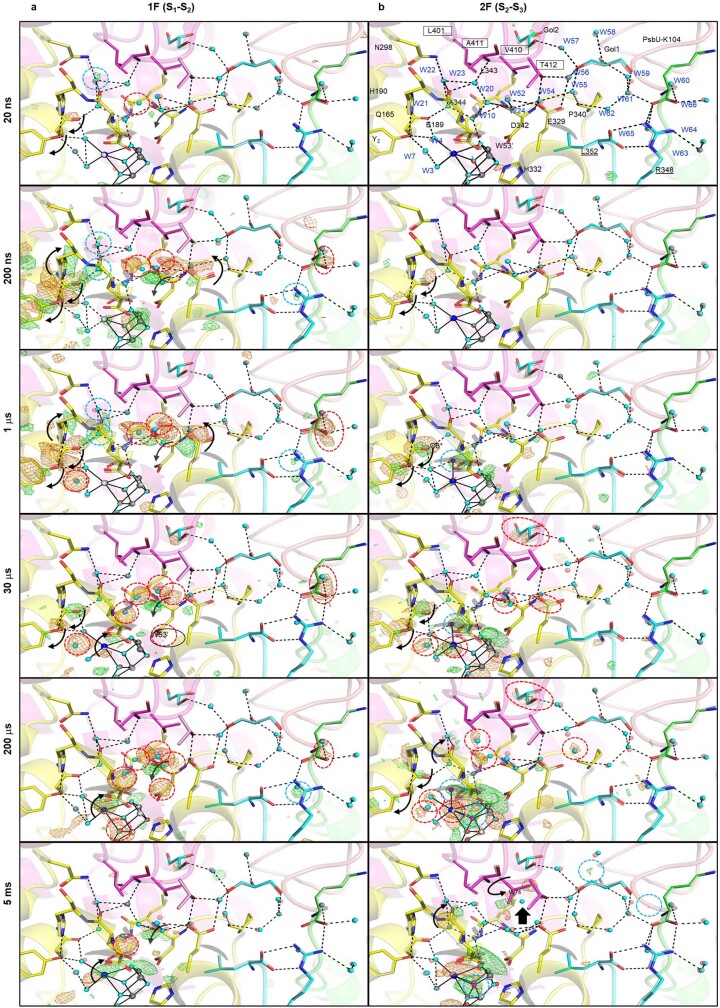
Structural dynamics at the O1 channel during S_1_–S_2_–S_3_ transitions. **a**,**b**, Structures of PSII at the O1 channel are superposed with *F*_obs_(1F) – *F*_obs_(dark) (**a**) and *F*_obs_(2F) – *F*_obs_(1F) (**b**) difference density maps contoured at +4.0*σ* (green) and −4.0*σ* (orange), with delay times from 20 ns to 5 ms. The intermediate structures of D1, D2, CP43, PsbU and PsbV proteins are depicted in yellow, cyan, magenta, green and pink, respectively. Water molecule W53 in the S_1_ state is only observable under cryo-temperature conditions (PDB codes: 3WU2 and 4UB6) and is not detectable at room temperature (PDB codes: 5WS5 and 7CJI). Because the protein environment around W53 is the same regardless of temperature, W53 is considered present but fluctuating. Therefore, in this study, we denote this invisible water as W53′.

We observed previously that 1F leads to the disorder of two water molecules—one in the O1 channel and the other in the O4 channel^6,25^. The current study reveals the dynamic behaviour of the water molecules in these channels. At Δ*t*1 = 30 μs to 5 ms, disorder of W10 and the concomitant movement of D1-D342 were observed, consistent with the previously solved structure of the S_2_ state^25^ (Extended Data Fig. 7a). At Δ*t*1 ≤ 200 μs, dynamic difference densities appear in the O1 channel. These span from a five-water cluster located near the OEC to the PsbU-K104–D2-R348 (PsbU is a subunit of PSII) salt bridge near the lumen (Extended Data Fig. 7a). Difference densities appear at Δ*t*1 = 20 ns on W20, W22 and the nearby D1-D342 main chain; these are likely to be induced by correlated movements of the neighbouring Y_Z_ and D1-Q165 (Extended Data Fig. 7a). As Y_Z_ and D1-Q165 move to a greater extent at Δ*t*1 = 200 ns and Δ*t*1 = 1 μs, the positive electron density on W22 increases and spreads to cover W21 and W22. Subsequently, Y_Z_ and D1-Q165 move backwards at Δ*t*1 = 30 μs to Δ*t*1 = 5 ms, and the difference densities vanish (Extended Data Fig. 7a; see Fig. 3a for a closer view). By contrast, the difference densities near the main chain of D1-D342 persist from Δ*t*1 = 20 ns to Δ*t*1 = 5 ms, indicating its shift throughout the S_1_–S_2_ transition. Furthermore, during Δ*t*1 = 20 ns to Δ*t*1 = 200 μs, movement of the D1-D342 main chain induces disorders or shifts of W20, W24, W52 and D1-E329, which are connected to D1-D342 by hydrogen bonds. At Δ*t*1 = 30–200 μs, a negative difference density arises on W53′, which is located in the cavity surrounded by OEC-O1, D1-E189, D1-E329, D1-H332 and D1-D342 (Extended Data Fig. 7a), indicating that W53′ becomes further disordered. Here, W53 in the S_1_ state is only observable under cryo-temperature conditions (PDB codes: 3WU2 (ref. ^2^) and 4UB6 (ref. ^3^)) but is not detectable at room temperature (PDB codes: 5WS5 (ref. ^4^) and 7CJI (ref. ^25^)). Therefore, we denote this invisible water as W53′ (Extended Data Fig. 1c and Extended Data Fig. 7a).

A negative difference density appears on the PsbU-K104 carboxy terminal at the O1-channel entrance during Δ*t*1 = 200 ns–200 μs and subsequently disappears by Δ*t*1 = 5 ms (Extended Data Fig. 7a). The PsbU-K104–D2-R348 salt bridge might function as a gate for the O1 channel, and the PsbU-K104 disorder implies the breakage or loosening of the salt bridge (Extended Data Fig. 7a), resulting in the opening of the gate and the entry of water into the giant cavity that houses Gol1, W55, W56, W59, W61 and W62 (Extended Data Fig. 7a).

**Fig. 4:**
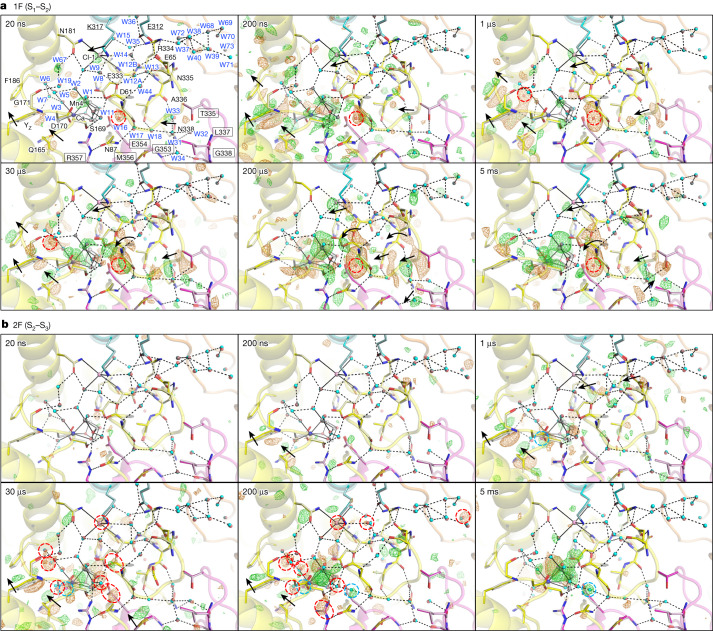
Structural dynamics at the O4 and Cl-1 channels during S_1_–S_2_–S_3_ transitions. **a**,**b**, Structures of PSII at the O4 and Cl-1 channels are superposed with *F*_obs_(1F) – *F*_obs_(Dark) (**a**) and *F*_obs_(2F) – *F*_obs_(1F) (**b**) difference density maps contoured at +3.5*σ* (green) and −3.5*σ* (orange) with delay times from 20 ns to 5 ms. The intermediate structures of D1, D2, CP43 and PsbO proteins are depicted in yellow, cyan, magenta and orange, respectively, and the colours of other atoms are the same as those in Figs. 1 and 2.

At Δ*t*2 = 20 ns to Δ*t*2 = 200 ns, there is no difference density in the O1 channel (Extended Data Fig. 7b). At Δ*t*2 = 1 μs, the most noticeable difference density occurs on O6* (Fig. 4b). When O6* is being prepared and translocated to O6 at Δ*t*2 = 30 μs to Δ*t*2 = 200 μs, negative difference densities appear on the main chains of D1-D342, D1-E329, W24, W52, W55 and Gol2, indicating that they are disordered during the O6* translocation. At Δ*t*2 = 5 ms, the disordered components become ordered again after the completion of the O6* translocation. In addition, paired positive and negative difference densities appeared around CP43-V410 (CP43 is a subunit of PSII), suggesting a rotation of the CP43-V410 side chain by 120° rather than a mere shift as proposed previously^6^ (Extended Data Fig. 7b). In conjunction with the CP43-V410 rotation, a partially occupied water designated as W74 emerges in the proximity of the pre-rotation conformation of CP43-V410 (Extended Data Figs. 5c and 7b). Furthermore, two small positive densities appear near Gol1 and PsbU-K104 at Δ*t*2 = 5 ms, which suggests that two new water molecules become ordered at the end of the O1 channel after O6 translocation, similar to the findings of a previous study^6^ (Extended Data Fig. 5c).

## Structural changes in the O4 channel

W16 is the second water in the O4 channel and is disordered after 1F (Fig. 4a). This disorder is maintained after 2F, until returning to a stable state after 3F (refs. ^5–7^). Disorder of W16 initiates at Δ*t*1 = 20 ns, increases progressively, and reaches a maximum at Δ*t*1 = 200 μs (Fig. 4a and Supplementary Table 3). The W16 disorder is expected to be influenced by the charge rearrangement that occurs at Δ*t*1 = 20–200 ns, the oxidation of Mn4 at Δ*t*1 = 1–30 μs and the stabilization of the remaining positive charge on the OEC at Δ*t*1 = 200 μs–5 ms (Fig. 3a). One potential explanation is that the alternation of Mn4 charge influences W11 through O4, leading to the disruption of the hydrogen bond between W11 and W16 and the W16 disorder. The W16 disorder further affects the hydrogen-bonding network at the O4 channel, leading to shifts of W18, W31, W33 and W34 from Δ*t*1 = 20 ns to Δ*t*1 = 5 ms (Fig. 4a). In addition, when the difference densities in the O4 channel are strongest at Δ*t*1 = 200 μs, the main chains of D1-R334-N335-A336 showed slight shifts towards the OEC. Movement of D1-D61 towards the OEC is also observed at Δ*t*1 = 30 μs–5 ms (Fig. 4a).

No noticeable difference densities are observed in the O4 channel at Δ*t*2 = 20 ns–1 μs. At Δ*t*2 = 30 μs, negative difference densities emerge on W11, CP43-E354 and D1-D61 (Fig. 4b), indicating their instability during this period. The nearby CP43-M356 appears to move toward this region (Fig. 4b), possibly to fill the space. At Δ*t*2 = 200 μs, W11 maintains its disorder, whereas CP43-E354, D1-D61 and CP43-M356 are restabilized (Fig. 4b). CP43-R357, which is located at a hydrogen-bonding distance to O4, becomes disordered at Δt2 = 200 μs. All of these residues and water molecules become ordered at Δ*t*2 = 5 ms (Fig. 4b). The oxidation of the OEC that takes place at Δ*t*2 = 30–200 μs could potentially contribute to the structural changes on W11, CP43-E354 and CP43-R357; all are directly connected to the OEC.

## Roles of Cl-1 in the S-state transitions

Difference densities near Cl-1 are observed at Δ*t*1 = 20 ns–5 ms; however, they fluctuate over time, in contrast to the nearly continuously growing difference densities observed on W16 (Fig. 4a and Supplementary Table 3). The difference densities near Cl-1 arise at Δ*t*1 = 20 ns, reach a maximum at Δ*t*1 = 200 ns, decline at Δ*t*1 = 1–30 μs, increase again at Δ*t*1 = 200 μs and finally decrease at Δ*t*1 = 5 ms. Considering the dynamics of the Y_Z_ area and the OEC at Δ*t*1 = 20 ns–5 ms, we hypothesize that the electrostatic effect of Y_Z_ and the OEC influences Cl-1, causing the fluctuation of the difference densities (Figs. 2a, 3a and 4a and Supplementary Table 3). The difference densities near Cl-1 are observed when Y_Z_ is oxidized to Y_Z_^•+^ at Δ*t*1 = 20 ns (Figs. 2a and 4a). These difference densities reach their peaks when Y_Z_^•+^ loses one proton, resulting in the formation of more oxidized Y_Z_^•^ at Δ*t*1 = 200 ns (Figs. 2a and 4a). Subsequently, difference densities near Cl-1 decrease during the reduction of Y_Z_^•^ at Δ*t*1 = 1 μs and Δ*t*1 = 30 μs. The disruption of the hydrogen-bonding network between Y_Z_ and Cl-1 could also contribute to the decreased signals (Figs. 2a and 4a). As the reduction of Y_Z_^•^ is completed and the OEC is oxidized to OEC^+^ at Δ*t*1 = 200 μs, the difference densities near Cl-1 increase again (Figs. 3a and 4a). The subsequently decreased difference density at Δ*t*1 = 5 ms could be attributable to a stabilization effect of the positive charge on the OEC. On the basis of these observations, Cl-1 might actively contribute to stabilizing the positively charged Y_Z_ and OEC during the S_1_–S_2_ transition.

Although paired positive and negative difference densities surrounding the two sides of Cl-1 were observed at Δ*t*1 = 20 ns–5 ms (some negative densities are weaker, which cannot be visible at the contour level ±3.5*σ*), a single negative difference density was observed overlaying Cl-1 at Δ*t*2 = 30 μs and Δ*t*2 = 200 μs (Fig. 4b and Supplementary Table 3). This indicates the disorder of Cl-1 during this time period after 2F (Fig. 4b), which is apparently different from the movements of Cl-1 observed after 1F. These differences show that Cl-1 has different roles in the S_1_–S_2_ and S_2_–S_3_ transitions, and the instability of Cl-1 after 2F might reflect the proton transfer along the Cl-1 channel as described below.

## Proton transfer through the Cl-1 channel

**Extended Data Fig. 8:**
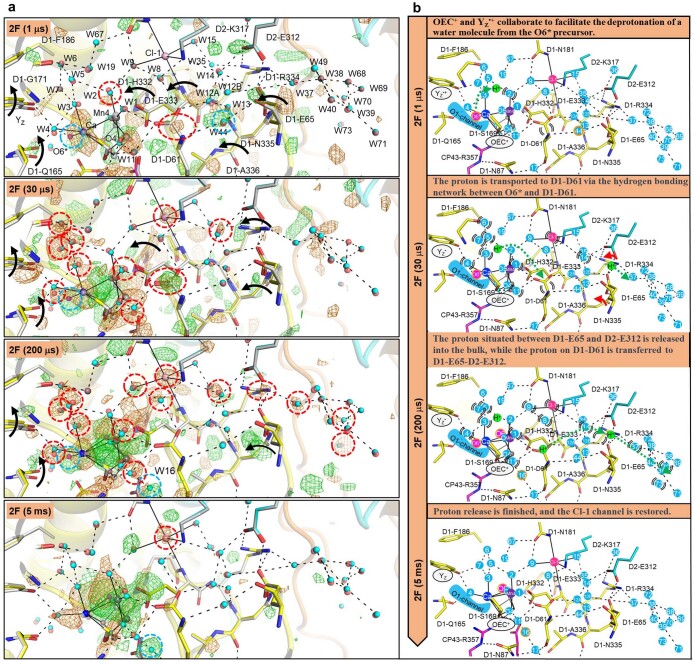
Structural changes at the Cl-1 channel and proton release pathways after 2F. **a**, Structures of PSII at Cl-1 channels superposed with *F*_obs_(2F) – *F*_obs_(1F) difference densities contoured at +3.0*σ* (green) and −3.0*σ* (orange), with delay times from 1 µs to 5 ms. The 1F model is depicted in grey, and intermediate structures of D1, D2, CP43 and PsbO proteins are depicted in yellow, cyan, magenta and orange, respectively. Water molecules at their ground states and intermediate states are depicted in red and cyan spheres, respectively. O6* and O6 are represented by magenta spheres. Black dotted lines represent hydrogen bonds. Black solid lines link the oxo-oxygen in the OEC and the ligand water to the metal ions, as well as ligand residues and water molecules to Cl-1. Black arrows indicate structural changes. Disordered water and residues are encircled by red dotted lines, and ordered water molecules in the intermediate structures are encircled by cyan dotted lines. **b**, Possible proton release pathways after 2F. Water molecules are depicted as cyan spheres, with their corresponding numbers labelled. Disordered water molecules, Cl-1 and residues are depicted with arched lines, and an orange outer ring around water molecules indicates that they became ordered. The red arrows indicate the movements of residues. The green dotted arrows indicate the movements of protons.

After 2F, structural changes in the Cl-1 channel are relatively small compared with those at other sites, and take place mainly between Δ*t*2 = 1 μs and Δ*t*2 = 200 μs (Fig. 4b and Extended Data Fig. 8a). At Δ*t*2 = 1 μs, W2 and D1-D61 become unstable, as indicated by the negative difference densities on them (Extended Data Fig. 8a), which might be attributable to the release of a proton from the precursor of O6* during this period. Concomitantly, W44 becomes transiently stable, probably as a result of the movement of D1-E65,which is connected to W44 (Extended Data Fig. 8a). Another structural change occurring at Δ*t*2 = 1 μs is the movement of the D1-H332 to D1-A336 backbones towards the OEC, which probably occurs owing to structural changes in the OEC. At Δ*t*2 = 30 μs, a larger number of water molecules in the hydrogen-bonding network become unstable (W1, W3-W7 and W11), and Cl-1 also starts to become unstable (Extended Data Fig. 8a). These structural changes imply that the proton is transferred to D1-D61 (Extended Data Fig. 8b). The movement of the D1-H332 to D1-A336 backbones is transmitted to the D1-R334 and D1-N335 side chains, leading to a shift of D1-R334 towards the OEC. This results in an instability of W14, and could potentially influence the gate between D1-E65, D2-E312 and D1-R334 (refs. ^8,17^). At Δ*t*2 = 200 μs, water molecules in the bulk region (W37, W70 and W73) also become unstable, in addition to the unstable water molecules near the OEC and Cl-1 (W1, W3–W5, W7–W9, W11 and W14) (Extended Data Fig. 8a), suggesting a possible proton transfer to the lumen. The instability of Cl-1 further increases at Δ*t*2 = 200 μs, and D1-R334 also exhibits instability (Extended Data Fig. 8a), presumably reflecting the pass of the proton^39,40^. By Δ*t*2 = 5 ms, the structural changes in the gate area, as well as the high mobilities of water molecules observed at Δ*t*2 = 200 μs, disappear entirely, and Cl-1 becomes ordered and returns to its original position (Extended Data Fig. 8). This indicates that the Cl-1 channel has been restored and reset to the subsequent S-state transition.

**Fig. 5:**
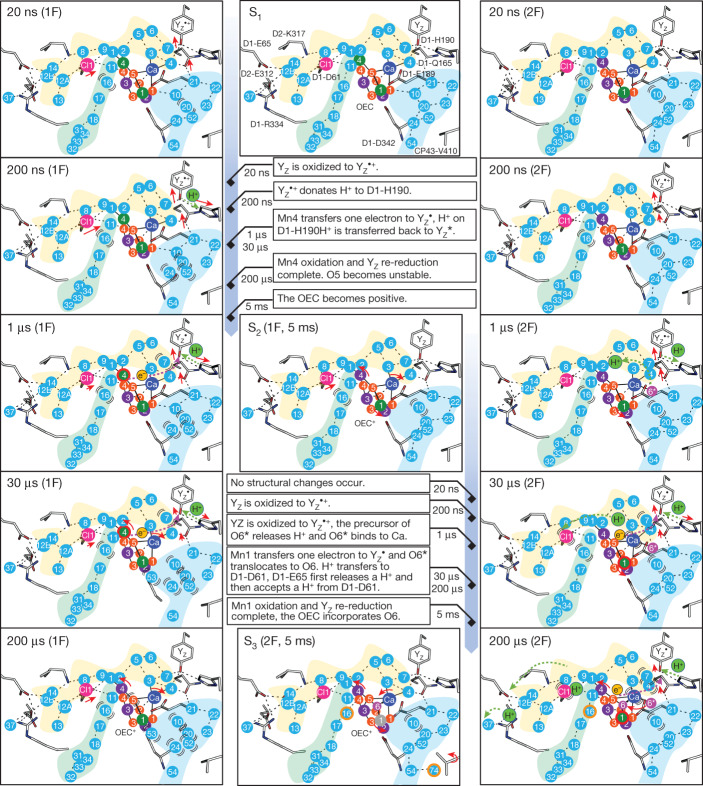
Schematic of events occurring during S_1_–S_2_–S_3_ transitions at the electron donor side. The small orange spheres correspond to O1–O5 and are numbered 1 to 5 in the OEC. O6* and O6 are shown as magenta spheres. The larger green, purple and grey spheres represent Mn1–Mn4 with labels 1 to 4. Specifically, the green spheres correspond to Mn(III), the purple spheres correspond to Mn(IV) and the grey sphere represents either Mn(III) or Mn(IV). A red outer ring of the spheres signifies that the Mn ion is undergoing oxidation. The Cl-1, O4 and O1 channels are depicted in yellow, light green and cyan backgrounds, respectively. Water molecules are depicted as cyan spheres, with their corresponding numbers labelled. Disordered water molecules and other disordered atoms are depicted with arched lines, and an orange outer ring of the water molecules indicate that they become ordered. The red arrows indicate the movements of residues and atoms; the length of the arrows roughly represents the travelled distance for Y_Z_ and Cl-1. The purple- and green-dotted arrows indicate the movements of electrons and protons, respectively. The proton transfer from Y_Z_ to D1-H190 takes place between 2F (1 µs) and 2F (30 µs), which was depicted at 2F (1 µs) owing to the absence of time points between 1 μs and 30 μs.

In conclusion, our time-resolved SFX experiments reveal the important roles of protein structural dynamics in electron transfer, water insertion, proton release and O–O bond formation in PSII. We summarize our results in a model presented in Fig. 5, and a detailed discussion is provided in the Supplementary Information.

## Methods

### Sample preparation

Samples of the PSII microcrystals were prepared as in the previous SFX studies conducted at room temperature^4,25^, with a few minor adjustments. In brief, cells of the thermophilic cyanobacterium *Thermosynechococcus vulcanus* were grown in a previously described medium^41–44^ in eight 5-l bottles, to a density of OD_730 __nm_ = 2.5–3.0, and collected as described previously^41–44^. The cells were resuspended in a buffer of 40 mM KH_2_PO_4_-KOH (pH 6.8) and 0.4 M mannitol, and treated with 1.21 g l^−1^ lysozyme (FUJIFILM Wako Pure Chemical Corporation) at 37 °C for 90 min with constant shaking. The treated cells were pelleted by centrifugation at 13,700*g* for 15 min, suspended in 25% (w/v) glycerol, 20 mM HEPES-NaOH (pH 7.0) and 10 mM MgCl_2_ (buffer A), and stored at −80 °C until use.

The frozen cells were thawed, to which ten folds of a buffer containing 30 mM HEPES-NaOH (pH 7.0) and 10 mM MgCl_2_ were added to disrupt the cells by freeze-thawing and osmotic shock. After centrifugation at 13,700*g* for 15 min, pelleted thylakoids were suspended in 5% (w/v) glycerol, 20 mM HEPES-NaOH (pH 7.0) and 10 mM MgCl_2_. Crude PSII particles were obtained from the thylakoids by a two-step solubilization with a detergent *N*,*N*-dimethyldodecylamine *N*-oxide (LDAO) (Sigma-Aldrich, 40236-250ML). In the first step, the thylakoids were treated with 0.16% (w/v) LDAO for 5 min on ice, and centrifuged at 43,200*g* for 60 min. The pellet obtained was suspended in buffer A, and treated with 0.27% (w/v) LDAO for 5 min again. The mixture was centrifuged at 100,000*g* for 1 h, and the supernatant was recovered. After the addition of 50 (w/v) polyethylene glycol (PEG) 1450 to a final concentration of 15%, crude PSII particles were recovered by centrifugation at 100,000*g* for 30 min, and resuspended in buffer A^41–44^.

The PSII crude particles were treated with 1.0% *n*-dodecyl-β-D-maltoside (β-DDM) (FUJIFILM Wako Pure Chemical Corporation, D316) for 5 min, and loaded onto a Q-Sepharose high-performance column (Cytiva) pre-equilibrated with 5% (w/v) glycerol, 30 mM MES-NaOH (pH 6.0), 3 mM CaCl_2_ and 0.03% β-DDM (buffer B) in a cooled chamber at 6 °C. The column was washed with eight to ten folds of the column volume of buffer B containing 170 mM NaCl, and eluted with a liner gradient of 12.5 folds of the column volume of 170–300 mM NaCl in buffer B. Elution peaks first appeared for PSII monomer, followed by PSII dimer and PSI monomer, among which PSII dimers were collected. The PSII dimers collected were diluted threefold by buffer B without DDM, and PEG 1450 was added to a final concentration of 13%. The PSII dimers were centrifuged at 100,000*g* for 30 min, and the pellet was suspended in buffer B without DDM and stored in liquid nitrogen until use^41–44^.

To make microcrystals of the PSII dimer, the sample was diluted with 20 mM MES-NaOH (pH 6.0), 40 mM MgSO_4_, 20 mM NaCl and 10 mM CaCl_2_, followed by additions of *n*-heptyl-β-D-thioglucopyranoside (HTG) (FUJIFILM Wako Pure Chemical Corporation, H015) and PEG 1450 to final concentrations of 0.85% (w/v) and around 5.50–5.75% (w/v), respectively, at a final concentration of 2.25 mg chlorophyll per ml (refs. ^4,6^). Microcrystals were grown in a 2.0-ml glass vial (J.G. Finneran Associates, 9800-1232), and 150 μl PSII dimer sample was put into each vial. After standing for 20–30 min at 20 °C, the solution was mixed gently and left to stand for another 10–30 min to allow the microcrystals to grow. In cases in which microcrystals did not appear or appeared in small numbers, the mixing-and-standing step was repeated until enough microcrystals appeared.

After the microcrystals appeared, they were allowed to grow to a maximum size of 100 μm in length for several hours to overnight, following which 150 μl of a crystal storage buffer containing 7% (w/v) PEG 1450, 20 mM MES-NaOH (pH 6.0), 20 mM NaCl, 10 mM CaCl_2_ and 0.85% (w/v) HTG was added to stop the growth of the microcrystals. After collection of the microcrystals, the supernatant was discarded, and the microcrystals were stored in the crystal storage buffer at 20 °C until the X-ray free electron laser (XFEL) experiments. It is important to store the microcrystals in the crystal storage buffer for more than 24 h to ensure high resolution, and they are stable in the crystal storage buffer for at least three days but not more than seven days^4,6^.

Before conducting the diffraction experiment, a 10 mM potassium ferricyanide solution was added to the PSII microcrystal solution under dim green light, and one pre-flash was given at 20 °C with a laser at a wavelength of 532 nm and an energy of 52 mJ cm^−2^. The microcrystals were subsequently transferred to 7% (w/v) PEG 1450, 20 mM MES-NaOH (pH 6.0), 20 mM NaCl, 10 mM CaCl_2_, 0.85% (w/v) HTG, 2% dimethyl sulfoxide (DMSO) and 10 mM potassium ferricyanide, and incubated for 10 min at 20 °C. The solution was finally replaced by a cryoprotectant solution containing 10% (w/v) PEG 1450, 10% (w/v) PEG monomethyl ether 5000, 23% (w/v) glycerol, 20 mM NaCl, 10 mM CaCl_2_, 0.85% (w/v) HTG, 2% DMSO and 10 mM potassium ferricyanide for six steps, with each step for 10 min at 20 °C (refs. ^4,6^).

After replacement of the solution with the cryoprotectant solution, PSII microcrystals were gently mixed with a vacuum grease of a nuclear power grade (Super Lube, 42150)^45^. The ratio of grease to microcrystals was 200 μl to 50 μl (obtained from 4–5 mg chlorophyll), and to avoid physical damage to the microcrystals, the mixing was conducted gently for 2 min. The mixture was exposed to air at 20 °C for around 30–60 min to dehydrate further, before being used for the diffraction experiments at room temperature in darkness^4^. The total time from cryoprotectant replacement to XFEL experiments was one to two hours.

### Diffraction experiment

The dark and 1F data, as well as the 1F and 2F time-delayed data, were collected in two independent experiments, resulting in a total of 14 experimental datasets (Extended Data Table 1). Unless otherwise stated, the experimental set-ups were identical for both beamtimes. Diffraction images were obtained using single-shot XFELs collected at the BL2 beamline in the SPring-8 Ångstrom Compact Free Electron Laser (SACLA)^46^. The parameters of the XFEL pulses were as follows: pulse duration 10 fs, energy 10 keV, beam size 3.0 μm (H) × 3.0 μm (W) and repetition rate 10 Hz (ref. ^4^). The PSII microcrystals were excited using pump lasers with the following parameters: pulse duration 6 ns (FWHM, Gaussian), energy 42 mJ cm^−2^, focused spot size 240 μm (top-hat), wavelength 532 nm and frequency rate 10 Hz (ref. ^4^). To ensure efficient excitation, one laser beam was split into two beams that focused on the same point of the sample from two different directions separated by an angle of 160° (ref. ^4^).

The injector containing the mixture of PSII microcrystals and grease was carefully inserted into a sample chamber, in which the mixture was ejected from the injector using liquid pressure, ultimately forming a micrometre-sized liquid stream^47,48^.

The sample flow rate is regulated by adjusting the fluid pressure in the injector. For the ‘dark’ sample, the flow rate is 1.99 μl min^−1^, whereas for the ‘light’ samples, it is 7.80 μl min^−1^. As described previously, by maintaining this flow rate, contamination from the prior lasers is effectively avoided^25^. The dark dataset was obtained by directly exposing the sample stream to XFELs, whereas the 1F and 2F datasets were acquired by illuminating the sample stream with the pump laser first, followed by exposure to the XFELs after a specified delay time Δ*t*. The values of Δ*t*1 and Δ*t*2 were set to 20 ns, 200 ns, 1 μs, 30 μs, 200 μs and 5 ms, respectively (Extended Data Fig. 1d). In addition, in the 2F time-delay experiment, the time interval between the first and second flash was set to 5 ms (Extended Data Fig. 1d), which is enough to fully transform the S_1_ state to the S_2_ state after 1F. The focal centres of the lasers and XFELs were the same for data with a Δ*t* of 20 ns–200 μs, but for data with a Δ*t* of 5 ms, the focal centres of lasers were set 60 μm higher than those of the XFELs to prevent the light-excited microcrystals from escaping the XFEL irradiations after a Δ*t* of 5 ms. Diffraction spots were recorded using a Rayonix MX300-HS detector, which was positioned 240 mm from the sample.

### Data processing

During the beamtime, we used Cheetah^49^ (https://github.com/keitaroyam/cheetah) and CrystFEL (v.0.6.3)^50,51^ to observe and analyse the diffraction images. The analyses provide hit rates, the number of indexed images and approximate resolutions for each dataset, which greatly aided us in devising an effective data-collection strategy. For the processing of diffraction images at the beamline, we at first used approximately 10,000 indexed diffraction images from lysozyme crystals to determine the beam centre and camera length accurately. These parameters were then supplied to CrytFEL for processing the PSII diffraction images. The PSII diffraction images were indexed with ‘indexamajig’, using the Dirax^50,51^ indexing method with unit-cell parameters of *a* = 124.7 Å, *b* = 229.89 Å, *c* = 285.5 Å, *α* = *β* = *γ* = 90° adopted from PDB code 5WS5 (ref. ^4^). The resulting individual intensities were merged using ‘process_hkl’ and the reflection data were evaluated using ‘compare_hkl’ (refs. ^50,51^).

After data collection, ‘cctbx.xfel’ was used for the indexing and integration of diffraction images, as well as for merging reflections^52,53^. The accuracy of the beam centre and camera length obtained from CrystFEL were verified by using the program ‘cspad.cbf_metrology’ (refs. ^52,53^). The PSII diffraction images were indexed and integrated using ‘dials.stills_process’ (ref. ^54^), incorporating the determined detector information and targeted unit-cell parameters mentioned above. Individual reflections were merged by the program ‘cxi.merge’ (refs. ^52,53^) with the post-refinement rs2 algorithm, and a filter based on the value of I/sigma was not applied so as to include weak signals at high resolutions. The average unit cell, calculated from all of the datasets collected in the same experiment, was used to merge each individual dataset once again. All datasets were processed to a resolution of 2.15–2.30 Å on the basis of the criteria of *CC*_1/2_ of around 50% (Extended Data Table 1).

### Structural refinement for the dark and 1F datasets

Molecular replacement for the dark data was performed using Phaser-MR from PHENIX^55^ with the PSII structure solved at 2.35-Å resolution and at room temperature (PDB code: 5WS5) as the search model, in which water molecules and the OEC were removed^4^. Next, rigid body refinement was applied to the resultant model for one cycle. Subsequently, the B factor was set to 20 for all atoms in the model, and the atomic coordinates and temperature factors of atoms were refined by ‘Phenix.refine’ in the resolution range of 2.15–20.0 Å, in conjunction with manual modifications by Coot^56^. We iteratively carried out reciprocal space refinement using ‘Phenix.refine’ and real-space refinement using Coot until the structures of residues and cofactors were confined. Then, the OEC and water molecules were added to the model. Geometric restraints of the OEC are based on the Mn_4_CaO_5_ cluster solved at 2.15 Å using microcrystals at cryo-temperature (PDB code: 6JLJ)^6^, with a loose distance restraint of *σ* = 0.06 Å on Mn–O and Ca–O distances, whereas no restraints were provided for the Mn–Mn and Mn–Ca distances. Any pre-existing water molecules exhibiting negative m*F*_o_-D*F*_c_ signals or lacking 2m*F*_o_-D*F*_c_ signals were removed from the model. New water molecules were constructed at the positions of positive spherical m*F*_o_-D*F*_c_ signals over 4*σ*, and these water molecules were examined after subsequent rounds of reciprocal and real-space refinements to confirm. Finally, a TLS refinement was applied.

For the refinement of the 1F model in the two-flash time-delay experiments, we assigned a single conformation to the OEC and ligands, considering that the geometry of the OEC does not differ much between S_1_ and S_2_ states. During the refinement process, the Mn–Mn and Mn–Ca distances were not restrained, whereas the distances of Mn–O and Ca–O were restrained to the values observed in the 1F model solved at 2.15 Å (PDB code: 6JLK)^6^, and refined with a loose restraint (*σ* = 0.06 Å). W16 was removed from the model owing to the emergence of a negative m*F*_o_-D*F*_c_ signal when W16 was present, even at low occupancy. Conversely, W10 was retained because its deletion resulted in a significant positive m*F*_o_-D*F*_c_ signal at the corresponding location.

### Difference-map calculations and structural refinement of intermediates

The phases obtained from the well-refined dark and 1F models were used to calculate isomorphous-difference Fourier maps between dark and 1F time-delayed data, and between 1F and 2F time-delayed data, respectively. Substantial difference densities were detected in the Q_A_–Q_B_, P680, Y_Z_ and OEC channel regions at each time point, with their locations dynamically varying over time (Figs. 1–4 and Extended Data Fig. 7). To refine the dynamic intermediate structures conveniently and effectively, we devised double conformations for all residues, water molecules and ligands within a spherical range of 20 Å centred on the Ca of the OEC and the non-haem iron, with A and B conformations corresponding to structures of the ground state and intermediate state, respectively. In this case, unstable water molecules and residues in the intermediate state were also built into the structures. Whether to preserve or delete these water molecules is decided by examining the m*F*_o_-D*F*_c_ signal. For example, in the case of W16, which became very unstable after 1F, building two conformations resulted in a strong negative signal on W16. Therefore, we deleted the B conformation of W16. On the other hand, for other unstable water molecules, such as W7 and W10, building two conformations did not result in a particularly strong negative m*F*_o_-D*F*_c_ signal, so their B conformations were preserved. Populations of S_*i*_ state in PSII crystals were estimated to be 0.4/0.6 for S_1_/S_2_ after 1F and 0.49/0.51 for S_2_/S_3_ after 2F, on the basis of flash-induced Fourier transform infrared (FTIR) measurements^4,6,57^. On the basis of these ratios, we constructed the 1F structure by adopting two conformations for those atoms or residues that showed structural changes between S_1_ and S_2_. The S_2_-state structure was refined against the density map, whereas the S_1_-state structure was taken from the dark structure solved in the present study. On the other hand, in the 2F data, the structure of PSII that does not advance to the S_3_ state is a mixture of S_1_ and S_2_. Owing to the small structural changes between S_1_ and S_2_, we fixed the structure to the S_2_ state for PSII that does not advance to the S_3_ state after 2F, and refined the S_3_-state structure against the density map. These assignments do not pose major problems for modelling the structures according to the densities obtained. We refined the *xyz* coordinates of the B conformation, followed by refining the B factors of both the A and the B conformation, and applied TLS refinement at last.

O6* was modelled as a water molecule with an occupancy of 0.51, without imposing artificial constraints on its distance to Ca and the nearby water molecules. The structures of the OEC containing O6 at Δ*t*2 = 200 μs and Δ*t*2 = 5 ms were investigated using three different O5–O6 distances: 1.9 Å, 2.2 Å and 2.4 Å, as indicated by theoretical calculations^37,58^. The optimal distance was determined by assessing the magnitude of the adjacent m*F*_o_-D*F*_c_ signals (Extended Data Fig. 6).

We need to point out that, although the XFEL data collected in the present study have a high quality, and the resolutions obtained are high, uncertainties exist with regard to the subtle structural changes that occur during S_1_–S_2_–S_3_ transitions, and it is important not to overinterpret the crystallographic data presented in this study.

### Estimation of errors in inter-atomic distances

To estimate the errors in the inter-atomic distances, we used the resampling method, creating ten substructures with reduced data multiplicity. Subsequently, we calculated the standard deviations of atom–atom distances within these ten substructures. We resampled our XFEL data by the jackknifing method^59^. We began with a dataset consisting of 100% images and created ten sub-datasets by merging 75% randomly selected images. Subsequently, we refined ten substructures against these sub-datasets. To initiate the refinement of the substructures, we used the well-refined structure derived from the 100% image dataset as our starting model, resetting the temperature factors of all atoms to 20 Å^2^ and applying simulated annealing. After this, we performed refinements on the rigid body, atom position coordinates, temperature factors and TLS. The standard deviations of atom–atom distances were calculated across the ten substructures, which were used as estimates of the errors associated with the corresponding atom–atom distances in the determined structures (Extended Data Fig. 3).

### Density functional theory calculations

An OEC model of the S_3_ state for density functional theory (DFT) calculations was constructed from the XFEL model (monomer A) of PSII (PDB code: 6JLL)^6^. This model comprises 408 atoms, including the inorganic Mn_4_CaO_5_ cluster, 4 terminal aqua/hydroxo ligands at Ca and Mn4, 15 crystal waters along with one extra hydroxide anion referred to as O6*, one chloride anion, and the following amino acid residues: D1-D61, D1-N87, Yz, D1-Q165, D1-S169, D1-D170, D1-N181, D1-V185, D1-F182 (backbone only), D1-E189, D1-H190, D1-N296, D1-N298 (fragment), D2-K317 (fragment), D1-H332, D1-E333, D1-A336, D1-H337, D1-D342, D1-A344 (C terminus), CP43-E354, CP43-R357, CP43-L401, CP43-V410 and CP43-A411. The revision made to the previous computational model^6,37^ involves augmenting it with the incorporation of five water molecules next to O6*, called a ‘water wheel’, along with four supporting amino acid residues (D1-N296, CP43-L401, CP43-V410 and CP43-A411). Geometric optimizations for the hydroxo form of O6* bound to the Ca site of (Mn^IV^)_3_Mn^III^CaO_5_ were carried out at multiplicity 14 (*M*_S_ = 13/2) using the B3LYP hybrid functional^60^ augmented with the D3 version of Grimme’s empirical dispersion correction and the Becke–Johnson damping function^61,62^, in combination with the Los Alamos (LANL2DZ) pseudopotential basis set for Ca and Mn and 6-31G(d) for all other atoms^63–66^. A crucial requirement for the production of meta-stable Ca^2+^-bound hydroxo form of O6*, as displayed in Extended Data Fig. 5a,b, is the absence of a Y_Z_ radical (Tyr_Z_–O^•^…^+^HN–His190), as the p*K*_a_ value of Ca^2+^-bound water (around 12.7 in aqueous solution)^67,68^ might be much higher than that of the histidine residue (6.0) (ref. ^69^), even within the protein environment.

### Reporting summary

Further information on research design is available in the Nature Portfolio Reporting Summary linked to this article.

## Online content

Any methods, additional references, Nature Portfolio reporting summaries, source data, extended data, supplementary information, acknowledgements, peer review information; details of author contributions and competing interests; and statements of data and code availability are available at 10.1038/s41586-023-06987-5.

### Supplementary information

Supplementary Information This file contains Supplementary Discussion, Supplementary References, Supplementary Tables 1–3 and Supplementary Fig. 1.

Reporting Summary

Peer Review File

Supplementary Video 1 Structural dynamics at Q_A_-Q_B_ region after the first flash.

Supplementary Video 2 Structural dynamics at Q_A_-Q_B_ region after the second flash.

Supplementary Video 3 Structural dynamics at the electron donor region after the first flash.

Supplementary Video 4 Structural dynamics at the electron donor region after the second flash.

## Figures and Tables

**Extended Data Table 1:**
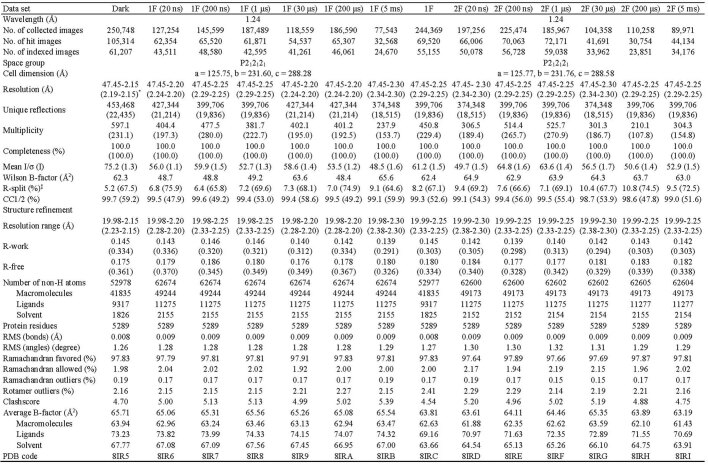
Data processing and structure refinement statistics

^*^Values in parentheses indicate those for the highest-resolution shells. ^‡^${R}_{{split}}=\sqrt{2}$ Σ|*I*_even_ – *I*_odd_|/Σ (*I*_even_ + *I*_odd_).

**Extended Data Table 2:**
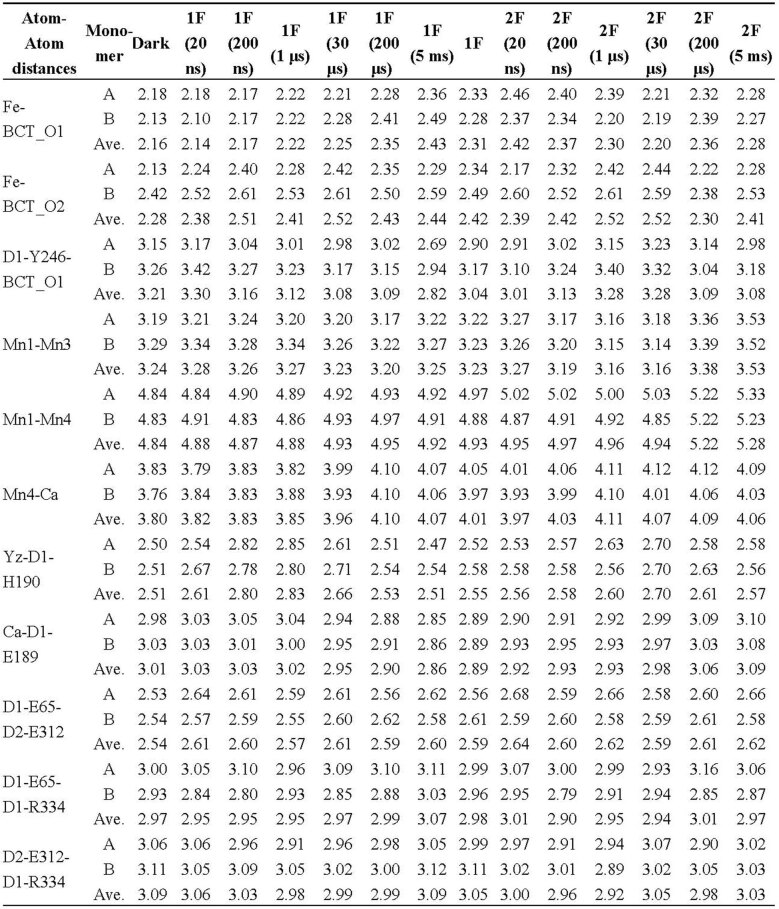
Atomic distances at various time points after 1F or 2F

## Data Availability

The atomic coordinates and structure factors have been deposited in the PDB under the following accession codes: 8IR5 for 0F (dark, ground state for the Δ*t*1 structures), 8IR6 for Δ*t*1 = 20 ns, 8IR7 for Δ*t*1 = 200 ns, 8IR8 for Δ*t*1 = 1 μs, 8IR9 for Δ*t*1 = 30 μs, 8IRA for Δ*t*1 = 200 μs, 8IRB for Δ*t*1 = 5 ms, 8IRC for 1F (ground state for the Δ*t*2 structures), 8IRD for Δ*t*2 = 20 ns, 8IRE for Δ*t*2 = 200 ns, 8IRF for Δ*t*2 = 1 μs, 8IRG for Δ*t*2 = 30 μs, 8IRH for Δ*t*2 = 200 μs and 8IRI for Δ*t*2 = 5 ms. All other data with a PDB code used in this study are from the PDB data bank.
