# Supplementary material for: Oxygen-evolving photosystem II structures during S1–S2–S3 transitions
Source: Nature. 2024 Jan 31;626(7999):670–7. doi: 10.1038/s41586-023-06987-5 (PMC10866707; doi:10.1038/s41586-023-06987-5)
Supplement: Supplementary file 1 — This file contains Supplementary Discussion, Supplementary References, Supplementary Tables 1–3 and Supplementary Fig. 1. [file 41586_2023_6987_MOESM1_ESM.docx]

**Supplementary Information for**

**Oxygen-evolving photosystem II structures during S_1_-S_2_-S_3_ transitions**

Hongjie Li, Yoshiki Nakajima, Eriko Nango, Shigeki Owada, Daichi Yamada, Kana Hashimoto, Fangjia Luo, Rie Tanaka, Fusamichi Akita, Koji Kato, Jungmin Kang, Yasunori Saitoh, Shunpei Kishi, Huaxin Yu, Naoki Matsubara, Hajime Fujii, Michihiro Sugahara, Mamoru Suzuki, Tetsuya Masuda, Tetsunari Kimura, Takanori Nakane, Tran Nguyen Thao, Shinichiro Yonekura, Long-Jiang Yu, Takehiko Tosha, Kensuke Tono, Yasumasa Joti, Takaki Hatsui, Makina Yabashi, Minoru Kubo, So Iwata, Hiroshi Isobe, Kizashi Yamaguchi, Michihiro Suga, Jian-Ren Shen

**This supplementary information contains**

**Supplementary Discussion**

**Supplementary References**

**Supplementary Tables 1-3**

**Supplementary Figure 1**

**Discussion**

A number of significant structural changes are found at both electron acceptor and donor sides following one or two flash illuminations. These changes reflect the transfer of one electron following each flash illumination, the release of one proton, and the incorporation of one water molecule into OEC during the S_1_-S_2_-S_3_ transitions. We discuss these structural changes in relation to the mechanism of electron transfer, proton release, and dioxygen formation in the following sections.

**Structural dynamics at the electron acceptor side**

Both the first and second flashes induce the transfer of an electron to the electron acceptor side. In physiological conditions, electrons from Q_A_^-^ will pass to Q_B_ directly, as the non-heme-iron (Fe^2+^) located between Q_A_ and Q_B_ has a high redox potential (*Em*) ^1-3^ and, therefore, does not participate in the electron transfer process directly. Because we added excess ferricyanide prior to the experiment, this will pre-oxidize the non-heme-iron to Fe^3+^. However, we gave a pre-flash before the pump-probe experiment, which will reduce Fe^3+^ to Fe^2+^. Due to the long time (1-2 hours) between the pre-flash and pump-probe experiment, the excess ferricyanide present in the solution will re-oxidize Fe^2+^ to Fe^3+^ again. This enabled Fe^3+^ to accept an electron from Q_A_^-^ following the first flash^1,4,5^. After the first flash, the appearance, weakening, and eventual disappearance of the difference densities near the Q_A_ binding site indicate the sequential process of electron transfer from P680 to Q_A_ and its subsequent release from Q_A_^-^ (Fig. 1a). The electron leaving Q_A_ is directed to the pre-oxidized non-heme-iron, resulting its reduction and the subsequent movement of the bound BCT at *Δt*1 = 5 ms (Fig. 1a). At *Δt*1 = 200 μs and 5 ms, there is a slight shift of the head group of Q_B_, which can be attributed to either i) the movement of BCT which may change the binding environment of Q_B_^6^, or ii) a small portion of electrons leaving Q_A_ reaching Q_B_, as previously observed^7^. In either case, the difference densities on the Q_A_ binding site at *Δt*1 = 20 ns - 200 ns are significantly stronger compared to that on the Q_B_ binding site at *Δt*1 = 5 ms (Fig. 1a), indicating that the Q_B_ binding site is predominantly occupied by Q_B_ rather than Q_B_^-^ after the first flash, which in turn indicates that the first electron transferred from O_A_ remained largely at the non-heme iron.

At a concentration of 10 mM ferricyanide, it takes 20 s to oxidize the non-heme-iron^4^. In our experiments, the interval between the first and second flashes is 5 ms (Extended Data Fig. 1d). Therefore, the non-heme-iron remains in the Fe^2+^ state when the second flash comes. Consequently, the second flash induces an electron that passes through Q_A_ and then to Q_B_ directly, which was evidenced by the difference densities on the Q_A_ binding site at *Δt*2 = 20 ns -200 μs and the positive difference density on the Q_B_ head at *Δt*2 = 5 ms (Fig. 1b). While the non-heme-iron does not accept electron, it may potentially facilitate the electron transfer from Q_A_^-^ to Q_B_, as it becomes transiently disordered at *Δt*2 = 200 μs (Fig. 1b).

The intensity of the difference density on the Q_A_ binding site is significantly weaker upon the formation of Q_A_^-^ by the second flash compared to that observed after the first flash (Fig. 1). This difference is likely attributed to the diminished excitation efficiency of P680 after the second flash. Furthermore, the intensity on the Q_A_ binding site undergoes a significant reduction at *Δt*1= 20 ns to 200 μs. In contrast, the weakening after the second flash is only marginal (Fig. 1). This observation demonstrates that the electron transfer from Q_A_^-^ to Fe^3+^ occurs at a faster rate compared to the transfer from Q_A_^-^ to Q_B_, which is well consistent with the respective time constant of 7 μs and 150 – 500 μs for the electron transfer from Q_A_^-^ to Fe^3+^ and Q_A_^-^ to Q_B_^1,7^.

**Structural dynamics at the electron donor side**

The structure changes at the electron donor side arise from a series of events involving electron transfer, proton transfer, and the transportation of water molecules. Fig. 5 presents a comprehensive image illustrating the events at the electron donor side during the S_1_-S_2_-S_3_ transitions.

The excitation of P680 and the subsequent formation of P680^•+^ occur in the picosecond time range, and corresponding structural changes were recently observed in the reaction center of a photosynthetic bacterium by the pump-probe SFX method^8^. Although our ns-ms delay time does not allow direct observation of this process, we found that the amino acid residues D1-Q165, Y_Z,_ and D1-F186 are shifted toward P680, together with the appearance of a positive difference density over Mg of P_D1_ at *Δt*1 = 200 ns (Fig. 2a). This suggests an electron transfer from Y_Z_ to P680^•+^. We may deduce that these structural changes occur before the electron transfer, as small negative difference densities already appeared at 20 ns following one flash (Fig. 2a), and it is reasonable to assume that these structural changes are required for the easy transfer of the electron from Y_Z_ to P680^•+^. This suggests that theoretical calculations of the electron transfer rate between the donor and acceptor based on their static distances may need to be modified, as there are subtle changes in the distances immediately before the electron transfer, although these changes are minor.

The processes that occurred in the region surrounding Y_Z_ in the ns-ms time range following the first and second flashes are essentially the same (Fig. 2, 5 and Extended Data Fig. 2). Following both 1F and 2F, Y_Z_ loses one electron to P680^•+^ and is accompanied by the proton transfer to D1-H190, resulting in the formation of Y_Z_^•^-D1-H190H^+^. Subsequently, OEC transfers one electron to Y_Z_^•^, leading to the reduction of Y_Z_^•^ and transfer of the proton from D1-H190H^+^ back to Y_Z_, ultimately restoring Y_Z_ to its original state (Fig. 5 and Extended Data Fig. 2c). However, the kinetics of these processes are slower after the second flash compared to the first flash (Fig. 2). The slower rate of Y_Z_ oxidation after the second flash is likely due to the remaining positive charge on OEC following one flash, which delays the electron transfer from Y_Z_ to P680^9^. The slower reduction of Y_Z_^•^ is attributed to the release of a proton prior to the donation of an electron by OEC^10^.

When Y_Z_ is in its reduced form in the dark and at *Δt* = 5 ms following one or two flashes, the distance between Y_Z_ and D1-H190 is 2.5 - 2.6 Å (Extended Data Fig. 3 and Extended Data Table 3), indicating the presence of a low barrier hydrogen bond (LBHB)^11-13^. These are consistent with the previous observations of Y_Z_-D1-H190 distances ranging from 2.40 Å to 2.60 Å in the S_1_-^11-13^, S_2_-^14,15,^ and S_3_-states^14,16^. However, when Y_Z_ is oxidized, and the proton on Y_Z_ is transferred to D1-H190, this distance becomes elongated, and the LBHB is converted to a standard hydrogen bond (Extended Data Fig. 3 and Extended Data Table 3).

W7 is a water molecule located close to Y_Z_, and its disorder occurs when the difference densities on Y_Z_ weaken following both 1F and 2F (Fig. 2). This disorder is probably caused by the re-gaining of one electron and proton by Y_Z_, as the time that the disorder occurs matches with these processes. After Y_Z_ receives one electron and restores to its original position, W7 becomes stable again.

**Structural changes of OEC**

The structural changes that occurred in OEC during the S_1_-S_2_ transition can be divided into three stages. In stage I (Δt1 = 20 ns – 200 ns), a charge rearrangement within OEC is triggered by the electrostatic effect of Y_Z_^•+^/Y_Z_^•^. In stage II (Δt1 = 1 μs – 30 μs), an electron is transferred from OEC to Y_Z_, and Mn4(III) is oxidized to Mn4(IV). Stage III (Δt1 = 200 μs – 5 ms) involves the rearrangement of OEC, which may be associated with a stabilizing effect of the remaining positive charge on OEC (Figs. 3a and 5). The charge rearrangement on OEC occurring within *Δt*1 = 200 ns may facilitate the subsequent electron transfer to Y_Z_ at *Δt*1=1 μs to 30 μs, which is completed by *Δt*1 = 200 μs (Figs. 3a and 5). During this process, OEC transforms to OEC^+^ due to the lack of proton release^10,17-19^. The unstable O5 at *Δt*1 = 200 μs is stabilized at *Δt*1 = 5 ms, and the slight outward movement of Mn1 may be caused by the rearrangement of OEC to stabilize the remaining positive charge (Figs. 3a and 5).

In the S_2_-state, the following changes occur in OEC compared with the S_1_-state: a slight opening of the geometry of OEC, retention of a positive charge, and the presence of two unstable water molecules near Ca and Mn4, respectively (Figs. 3a and 5). Following the second flash, the oxidation of Y_Z_ and the retained positive charge on OEC produces a 2+ charged state around OEC, which is unstable and needs to deprotonate unstable water near Ca, resulting in a new OH^-^ ion O6* bound to Ca to neutralize OEC^+^ (Figs. 3b and 5). Subsequently, OEC transfers one electron to Y_Z_, consistent with the subsequent release of a proton and electron transfer, as reported in numerous studies^7,10^. As OEC loses an electron to Y_Z_, Mn1(III) in OEC is oxidized to Mn1(IV), attracting O6*, the OH^-^ bound to Ca, to transfer to the position of O6. This illustrates that, for the electron transfer from OEC to Y_Z_ after the second flash, a water molecule (O6*) needs to bind to an intermediate position near Ca and be deprotonated. After the second flash, OEC still retains one positive charge, and the geometry of OEC opens further. These changes are expected to contribute to the release of the di-oxygen and the refilling of O5 in the subsequent S_3_-(S_4_)-S_0_ transitions.

Regarding the transfer pathway of O6*, the occurrence of O6* near Ca at *Δt*2 = 1 μs - 200 μs may not be consistent with a substrate water molecule entering the OEC through a carousel rearrangement around Mn4^20,21^. The pathway for the water molecules to reach O6 via W4 and W3 has been proposed by numerous studies^22-25^. However, W3 is relatively stable when the translocation of O6* occurs at *Δt*2 = 200 μs, so we consider that W3 may not participate in the direct translocation of O6*. The remaining pathway is a route for O6* to cross the barrier between D1-E189 and Ca, reaching the position of O6. There are three possible reasons supporting this scenario. First, at *Δt*2 = 200 μs, the Ca-D1-E189 distance may transiently increase during the O6* passage. Second, O6* is an OH^-^ ion requiring less space. Third, during the translocation process, O6* may further lose a proton to D1-E189, resulting in a Mn1(III)-O^•^ species as predicted by theoretical calculations^26^. The O5-O6 coupling mechanism has been extensively discussed^14-16,22,26-29^. The structure of OEC at *Δt*2 = 200 μs shows more features of the oxyl-oxo species, with an O5-O6 distance of 1.9 Å. However, the distance of O5-O6 became more diverse with no virtual differences in the range of 1.9-2.4 Å at *Δt*2 = 5 ms. It is possible that the current resolution is not high enough to determine the distance accurately. However, we have defined the distance of O5-O6 as 1.9 Å at cryo-temperature^14^. It may be possible that the oxyl/oxo and hydroxyl/oxo species are present as a mixture at room temperature, and both are able to proceed to the next S-state to evolve dioxygen.

After 1F, the oxidation of Mn4 triggers the outward movement of Ca and Mn4 from the OEC at *Δt*1 = 30 μs – 5 ms (Fig. 3a). The movement of Ca leads to the displacement of D1-E189, resulting in a shorter Ca-D1-E189 distance and the destabilization of W10 (Figs. 3a, 4a, 5, and Extended Data Fig. 3). Following the second flash, OEC^+^ and Y_Z_^•+^/ Y_Z_^•^ collectively lead to the deprotonation of the unstable W10, converting it to O6*, which binds to Ca and neutralizes the OEC^+^ (Fig. 3b). Subsequently, the oxidation by Y_Z_^•+^/ Y_Z_^•^ transforms Mn1(III) to Mn1(IV), causing translocation of O6* to O6, with Ca serving as the pivot point (Fig. 3b). Once the translocation of O6 is complete, Ca moves towards the center of OEC and the Ca-E189 is restored as in the S_1_-state (Fig. 3b). Water molecules present near CP43-V410 may then migrate to the position of W10. These observations indicate that Ca plays a vital role in the transfer of water molecules in the S_1_-S_2_ and S_2_-S_3_ transitions, consistent with that reported previously^30^.

**Structural changes in the O1-, O4- and Cl-1 channels**

The structural dynamics observed within the O1-channel after 1F and 2F indicate high mobility of water molecules within this channel (Extended Data Fig. 7)^14,22,27,30^. Following 1F, the O1-channel exhibits a wide range of diverse structural dynamics, including various behaviors of water molecules in the 5-water cluster, increased instability of the already unstable W53', and the instability of amino acid residues that form salt bridges at the lumenal exit of the O1-channel (Extended Data Fig. 7a). Most of these structural changes are transient, and as both Y_Z_ and OEC stabilize at 5 ms, most of the water molecules and residues in the O1-channel return to their original positions, except for W10, which remains unstable due to the movement of the Ca ion (Fig. 3a and Extended Data Fig. 7a). The structural changes in the O1-channel following 2F appear relatively more minor compared to those following 1F (Extended Data Fig. 7). This could be attributed to either more minor structural changes in the Y_Z_ area or the dominant allocation of the driving force towards deprotonation of the O6* precursor (Figs. 2 and Extended Data Fig. 7). Only during the ongoing translocation of O6* and after its completion, the neighboring residues and water molecules show some structural changes (Extended Data Fig. 7b). We note that when the translocation of O6* has finished, and Ca ion is still moving towards the center of OEC at 5 ms, there is no immediate replenishment of W10, as indicated by the absence of a positive difference density on W10 (Extended Data Fig. 7b). Nevertheless, a water molecule emerges near CP43-V410 following its rotation (Extended Data Fig. 5c and Extended Data Fig. 7b). This water molecule may eventually migrate to the position of W10 over a longer period or refill W10 after subsequent flashes. Moreover, the presence of two positive difference densities near Gol1 also suggests the emergence of new water molecules in the O1-channel (Extended Data Fig. 7b). However, the presence of a glycerol molecule in the O1-channel may differentiate the process of water delivery through the O1-channel in its natural state from its behavior in the crystal.

In the O4 channel, the instability of W16 begins at *Δt*1 = 20 ns and continues until *Δt*1 = 5 ms (Fig. 4a). This duration excludes the possibility that the instability of W16 is solely due to the oxidation of Mn4 or that O4 channel serves as the proton acceptor site in the S_1_-S_2_ transition as proposed previously^14^, as OEC remains unoxidized at *Δt*1 = 20 ns - 200 ns (Figs. 2a, 3a and 5). The instability of W16 may be attributed to changes in the charge distribution within OEC which involves charge rearrangement occurring between *Δt*1 = 20 ns – 200 ns, Mn4 oxidation taking place between *Δt*1 = 1 μs – 30 μs, and the stabilization of positive charges within OEC between *Δt*1 = 200 μs – 5 ms (Fig. 5). One plausible explanation is that changes in the charge distributions in OEC affect the hydrogen-bond between O4 and W11, which consequently disrupts the hydrogen-bond between W11 and W16, ultimately leading to the instability of W16. At *Δt*2 = 200 μs – 5 ms, W16 partially stabilizes again, possibly because OEC incorporates O6, which alters the charge distribution within OEC and consequently leads to the stability of W16 (Fig. 4b)

After 1F, the positive charges on Y_Z_ and OEC directly influence the movement of Cl-1 towards OEC and Y_Z_. Namely, Cl-1 plays a specific role in stabilizing the positive charges on Y_Z_ and OEC. Therefore, other substituents such as NO_3_^-^ and Br^-^ may also facilitate the smooth transition from S_1_ to S_2_ state^31^. This accounts for why the depletion of Cl^-^ does not affect the oxidation of Mn in the S_1_-S_2_ transition^32^. However, during the S_2_-S_3_ transition, the instability of Cl-1 between *Δt*2 = 30 μs - 200 μs indicates that Cl-1 may directly participate in the proton release. The removal or substitution of Cl-1 may lead to the collapse of nearby hydrogen-bonding networks, thereby preventing the release of protons and subsequent oxidation of OEC and entry of water molecules. As a result, PSII loses its functionality. These observations explain why Cl^-^ ions can be absent during the S_1_-S_2_ transition, but its absence inhibits the S_2_-S_3_ transition^32^.

The structural changes of the Cl-1 channel after 2F are apparently different from those after 1F and provide important clues to the proton release process in the S_2_-S_3_ transition. We identified a proton release pathway starting from O6*, passing through W1-W4, D1-D61, and finally reaching D-E65. The precursor of O6* may undergo deprotonation at *Δt*2 = 1 μs and the proton possibly travels from W4-W3-W5-W2-W1 to D1-D61 within *Δt*2 = 30 μs. At *Δt*2 = 200 μs, the proton on D1-E65 is released to the bulk under the influence of the movements of D1-R334 and D1-N335, with protons from D1-D61 being replenished onto D1-D65 (Fig. 5 and Extended Data Fig. 8). Cl-1 may play a role in maintaining the hydrogen-bonding network within the Cl-1 channel.

Yano and colleagues reported that D1-E65 rotates ~50° to open the ionic gate at 150 μs and then rotates back to close the gate at 250 μs after the second flash^27^, which are suggested to correspond to the proton release and refill processes. However, our structure showed that the distances between D1-E65, D1-R334, and D2-E312 do not change significantly following 2F (Extended Data Table 2). Proton transfer in the hydrogen-bonding network is likely achieved through the proton-hopping mechanism (Grotthuss mechanism^33^), facilitated by rearrangement of the hydrogen-bonding network, as observed in our study (Extended Data Fig. 8). Additionally, they observed an m*F*o-m*F*c signal indicative of a new water conformation at 250 μs and declining at 400 μs. However, this signal is consistently present at all time points in our structures. These signals indicate an alternative water conformation not accounted for in their structures. We refer to these water conformations as W12A and W12B.

In the current study, we show how two flashes work together to deprotonate a water molecule in the O1-channel, making it bound to OEC and preparing a state to produce di-oxygen (Fig. 5). In addition, as OEC after the second flash maintains OEC^+^, it is predictable that a proton will be released first when Y_Z_^•+^ is produced after the third flash, followed by the production of di-oxygen, release of another proton, and refill of O5.

**Existence of the O6 atom in the 2F structure**

Wang et al. employed XFEL data acquired initially by M. Ibrahim et al. to calculate the 2F-1F and 2F-Dark difference *Fourier* maps^22,34^. The analysis revealed a positive difference density peak near O6 or Ox^22^ located at distances of approximately 1.1 Å from O6 and 1.6 Å from O5. Wang et al. postulated a discrepancy between the positions of O6 and this positive difference density. Considering the presence of negative difference density adjacent to O5 in the vicinity of Mn4, Wang et al. proposed that the displacement of O5 might have contributed to the observed positive density near O6 rather than the insertion of a new water molecule. Subsequent refinements of the 2F data using a 1F single-conformation model, in which O5 moves by 0.5 Å and O6 is lacking, yielded noise-level m*F*o-D*F*c signals on OEC. This observation illustrated that the current 2F data^22^ can be adequately explained by the movement of O5 without invoking the insertion of O6.

However, in the current study, the *F*_o_^2F(5ms)^-*F*_o_^1F^ difference *Fourier* map contoured at ±7.0 σ reveals that the new O6 atom is not located at the center of the adjacent positive density (with a peak at +9 σ), but rather at the periphery of this approximate spherical positive density (Supplementary Fig. 1a). The distances from this positive peak to O5 and O6 are 1.9 Å and 0.4 Å, respectively (Supplementary Fig. 1b), suggesting that the positive difference density is more likely derived from the presence of O6. We can explain why O6 does not coincide perfectly with the center of the positive difference density as follows. There is a negative difference density on the opposing side of the positive difference density for Ca (with a peak at -4.7 σ) (Supplementary Fig. 1a). This pair of positive and negative difference densities can be interpreted as the inward movement of Ca toward the OEC and the positive peak near O6 may arise from a combination of O6 insertion and Ca movement.

To validate the location of O5 and O6, we employed Polder omit maps, wherein either O6 (Supplementary Fig. 1c, left panel) or both O5 and O6 in the 2F structure (Supplementary Fig. 1c, right panel) were omitted. The resultant maps revealed that O6 was located at the center of the peak. Moreover, the elliptical shape of the peak when both O5 and O6 were omitted strengthens the idea that two atoms, O5 and O6, occupy their positions, respectively, instead of a sole O5 atom in the 2F (5 ms) data.

Furthermore, when we reproduce electron densities using the methodology employed by Wang et al.^34^, in which the OEC consists of a single O5 model, residual positive *F*_o_-*F*_c_ peak appeared near the O6 site, with a peak height of 3.45 σ (Supplementary Fig. 1d). Because our analysis in which OEC contains both O5 and O6 atoms did not show such residual densities, we consider that both our interpretation and crystallographic analysis are valid. If we assume a single O5 model, distances from the O5 atom to Mn1, Mn3, and Mn4 were refined to be 2.65 Å, 2.04 Å, and 2.48 Å, respectively, based on the present structure (they are 2.63, 2.25, and 2.54 Å in Wang et al.^34^, respectively). These distances are too long and suggest that the O5 no longer has covalent bonds, at least with Mn1 and Mn4 atoms. This would disagree with the Mn valances of this structure predicted by several other approaches, including spectroscopic measurements. The structure is also inconsistent with general chemistry.

In summary, we consider that the insertion of O6 into OEC following 2F is the most appropriate scenario based on the analysis of *F*_o_^2F(5ms)^-*F*_o_^1F^ difference *Fourier* maps and the Polder omit maps.

**References (Supplementary Discussion and Supplementary Table 1)**

1. Petrouleas, V. & Diner, B. A. Identification of Q400, a high-potential electron acceptor of photosystem II, with the iron of the quinone-iron acceptor complex. *Biochim. Biophys. Acta -Bioenerg.* **849**, 264-275 (1986).
2. Krieger, A., Rutherford, A. W. & Johnson, G. N. On the determination of redox midpoint potential of the primary quinone electron acceptor, Q_A_, in photosystem II. *Biochim. Biophys. Acta -Bioenerg.* **1229**, 193-201 (1995).
3. Kato, Y., Nagao, R. & Noguchi, T. Redox potential of the terminal quinone electron acceptor Q_B_ in photosystem II reveals the mechanism of electron transfer regulation. *Proc. Natl. Acad. Sci. USA* **113**, 620-625 (2016).
4. Hienerwadel, R. & Berthomieu, C. Bicarbonate binding to the non-heme iron of photosystem II, investigated by Fourier transform infrared difference spectroscopy and ^13^C-labeled bicarbonate. *Biochemistry* **34**, 16288-16297 (1995).
5. Noguchi, T. & Inoue, Y. Identification of Fourier transform infrared signals from the non-heme iron in photosystem II. *J. Biochem.* **118**, 9-12 (1995).
6. Shevela, D., Eaton-Rye, J. J., Shen, J.-R. & Govindjee. Photosystem II and the unique role of bicarbonate: a historical perspective. *Biochim. Biophys. Acta* **1817**, 1134-1151 (2012).
7. Noguchi, T., Suzuki, H., Tsuno, M., Sugiura, M. & Kato, C. Time-resolved infrared detection of the proton and protein dynamics during photosynthetic oxygen evolution. *Biochemistry* **51**, 3205-3214 (2012).
8. Dods, R. *et al.* Ultrafast structural changes within a photosynthetic reaction centre. *Nature* **589**, 310-314 (2020).
9. Diner, B. A. & Britt, R. D. in *Photosystem II: The Light-Driven Water:Plastoquinone Oxidoreductase* (eds Thomas J. Wydrzynski, Kimiyuki Satoh, & Joel A. Freeman), 207-233 (Springer Netherlands, 2005).
10. Klauss, A., Haumann, M. & Dau, H. Seven steps of alternating electron and proton transfer in photosystem II water oxidation traced by time-resolved photothermal beam deflection at improved sensitivity. *J. Phys. Chem. B* **119**, 2677-2689 (2015).
11. Umena, Y., Kawakami, K., Shen, J.-R. & Kamiya, N. Crystal structure of oxygen-evolving photosystem II at a resolution of 1.9 Å. *Nature* **473**, 55-60 (2011).
12. Saito, K., Shen, J.-R., Ishida, T. & Ishikita, H. Short hydrogen bond between redox-active tyrosine y(z) and D1-His190 in the photosystem II crystal structure. *Biochemistry* **50,** 9836-9844 (2011).
13. Suga, M. *et al.* Native structure of photosystem II at 1.95 Å resolution viewed by femtosecond X-ray pulses. *Nature* **517**, 99-103 (2015).
14. Suga, M. *et al.* An oxyl/oxo mechanism for oxygen-oxygen coupling in PSII revealed by an x-ray free-electron laser. *Science* **366**, 334-338 (2019).
15. Li, H. *et al.* Capturing structural changes of the S_1_ to S_2_ transition of photosystem II using time-resolved serial femtosecond crystallography. *IUCrJ* **8**, 431-443 (2021).
16. Suga, M. *et al.* Light-induced structural changes and the site of O=O bond formation in PSII caught by XFEL. *Nature* **543**, 131-135 (2017).
17. Lavergne, J. & Junge, W. Proton release during the redox cycle of the water oxidase. *Photosyn. Res.* **38**, 279-296 (1993).
18. Schlodder, E. & Witt, H. T. Stoichiometry of proton release from the catalytic center in photosynthetic water oxidation: reexamination by a glass electrode study at pH 5.5–7.2. *J. Biol. Chem.* **274**, 30387-30392 (1999).
19. Suzuki, H., Sugiura, M. & Noguchi, T. Monitoring proton release during photosynthetic water oxidation in photosystem II by means of isotope-edited infrared spectroscopy. *J. Amer. Chemi. Soc.* **131**, 7849-7857 (2009).
20. Askerka, M., Wang, J., Vinyard, D. J., Brudvig, G. W. & Batista, V. S. S_3_ state of the O_2_-evolving complex of photosystem II: Insights from QM/MM, EXAFS, and femtosecond X-ray diffraction. *Biochemistry* **55**, 981-984 (2016).
21. Kawashima, K., Takaoka, T., Kimura, H., Saito, K. & Ishikita, H. O_2_ evolution and recovery of the water-oxidizing enzyme. *Nat. Commun.* **9**, 1247 (2018).
22. Ibrahim, M. *et al.* Untangling the sequence of events during the S_2_ --> S_3_ transition in photosystem II and implications for the water oxidation mechanism. *Proc. Natl. Acad. Sci. USA* **117**, 12624–12635 (2020).
23. Bovi, D., Narzi, D. & Guidoni, L. The S_2_ state of the oxygen‐evolving complex of photosystem II explored by QM/MM dynamics: spin surfaces and metastable states suggest a reaction path towards the S_3_ state. *Angew. Chem. Int. Ed. Engl.* **125**, 11960-11965 (2013).
24. Askerka, M., Brudvig, G. W. & Batista, V. S. The O_2_-evolving complex of photosystem II: Recent insights from quantum mechanics/molecular mechanics (QM/MM), extended X-ray absorption fine structure (EXAFS), and femtosecond X-ray crystallography data. *Acc. Chem. Res.* **50**, 41-48 (2017).
25. Kim, C. J. & Debus, R. J. One of the substrate waters for O_2_ formation in photosystem II is provided by the water-splitting Mn_4_CaO_5_ cluster's Ca_2+_ ion. *Biochemistry* **58**, 3185-3192 (2019).
26. Isobe, H., Shoji, M., Suzuki, T., Shen, J.-R. & Yamaguchi, K. Spin, valence, and structural isomerism in the S_3_ state of the oxygen-evolving complex of photosystem II as a manifestation of multimetallic cooperativity. *J. Chem. Theory Comp.* **15**, 2375-2391 (2019).
27. Hussein, R. *et al.* Structural dynamics in the water and proton channels of photosystem II during the S_2_ to S_3_ transition. *Nat. Commun.* **12**, 6531 (2021).
28. Bondar, A. N. & Dau, H. Extended protein/water H-bond networks in photosynthetic water oxidation. *Biochim. Biophys. Acta* **1817**, 1177-1190 (2012).
29. Yamaguchi, K. *et al.* Geometric, electronic and spin structures of the CaMn_4_O_5_ catalyst for water oxidation in oxygen-evolving photosystem II. Interplay between experiments and theoretical computations. *Coord. Chem. Rev.* **471**, 214742 (2022).
30. Nakamura, S., Ota, K., Shibuya, Y. & Noguchi, T. Role of a water network around the Mn_4_CaO_5_ cluster in photosynthetic water oxidation: A Fourier transform infrared spectroscopy and quantum mechanics/molecular mechanics calculation study. *Biochemistry* **55**, 597-607 (2016).
31. Wincencjusz, H., Yocum, C. F., & Van Gorkom, H. J. Activating anions that replace Cl^-^ in the O_2_-evolving complex of photosystem II slow the kinetics of the terminal step in water oxidation and destabilize the S_2_ and S_3_ states. *Biochemistry* **38,** 3719-3725 (1999).
32. Wincencjusz, H., van Gorkom, H. J. & Yocum, C. F. The photosynthetic oxygen evolving complex requires chloride for its redox state S_2_→ S_3_ and S_3_→ S_0_ transitions but not for S_0_→ S_1_ or S_1_→ S_2_ transitions. *Biochemistry* **36**, 3663-3670 (1997).
33. Agmon, N. The Grotthuss mechanism. *Chem. Phys. Lett.* **244,** 456–462 (1995).
34. Wang, J., Armstrong, W. H. & Batista, V. S. Do crystallographic XFEL data support binding of a water molecule to the oxygen-evolving complex of photosystem II exposed to two flashes of light? *Pro. Natl. Acad. Sci.* **118,** e32023982118 (2021).
35. Shoji, M. *et al.* Large-scale QM/MM calculations of hydrogen bonding networks for proton transfer and water inlet channels for water oxidation—Theoretical system models of the oxygen-evolving complex of Photosystem II. *Adv. Quant. Chem.* **70,** 325-413 (2015).

**Supplementary Table 1** | Numbering of water molecules in the O1-, O4 and Cl-1-channels. The letters preceding the slash denote the chain name, and "NA" indicates the absence of that water molecule in the structure.

| Current study | QM/　MM^35^ | This study (8IR5) | 3WU2^11^ | 4UB6^13^ | 6jlj^14^ | 7COU^15^ | 7RF1^27^ | Number  in Hussein et al.^27^ |
| --- | --- | --- | --- | --- | --- | --- | --- | --- |
| 1 | 1 | A/523 | A/595 | A/608 | A/567 | A/575 | A/515 | 1 |
| 2 | 2 | A/578 | A/624 | A/607 | A/588 | A/570 | A/555 | 2 |
| 3 | 3 | A/620 | A/569 | A/569 | A/641 | A/619 | A/603 | 3 |
| 4 | 4 | A/511 | A/544 | A/568 | A/525 | A/505 | A/525 | 4 |
| 5 | 5 | A/615 | A/509 | A/557 | A/644 | A/627 | A/622 | 24 |
| 6 | 6 | A/603 | A/567 | A/510 | A/633 | A/589 | A/575 | 23 |
| 7 | 7 | A/532 | A/564 | A/570 | A/508 | A/517 | A/541 | 25 |
| 8 | 8 | D/527 | A/621 | A/559 | D/514 | D/538 | A/512 | 21 |
| 9 | 9 | A/579 | A/609 | A/560 | A/565 | A/598 | A/550 | 22 |
| 10 | 10 | A/565 | A/597 | A/601 | A/507 | A/543 | A/612 | 26 |
| 11 | 11 | A/527 | A/511 | A/567 | A/523 | A/515 | A/519 | 19 |
| 12 | 12 | A/514 | A/593 | A/564 | A/518 | A/507 | A/534 | 40 |
| 13 | 13 | A/623 | A/553 | A/596 | A/629 | A/611 | A/630 | 42 |
| 14 | 14 | D/567 | D/586 | D/604 | D/578 | D/549 | D/567 | 41 |
| 15 | 15 | D/581 | D/531 | D/578 | D/570 | D/558 | D/554 | 60 |
| 16 | 16 | G/268 | C/633 | C/665 | C/699 | C/673 | - | - |
| 17 | 17 | A/552 | A/605 | A/542 | A/566 | A/544 | A/535 | 48 |
| 18 | 18 | A/541 | A/516 | A/546 | A/562 | A/537 | A/557 | 49 |
| 19 | 19 | A/544 | A/572 | A/556 | A/541 | A/523 | A/510 | 62 |
| 20 | 20 | A/559 | A/508 | A/547 | A/595 | A/580 | A/505 | 27 |
| 21 | 21 | A/599 | A/623 | A/588 | A/627 | A/599 | A/619 | 28 |
| 22 | 22 | A/612 | A/562 | A/543 | A/623 | A/616 | A/576 | 29 |
| 23 | 23 | A/520 | A/576 | A/536 | A/543 | A/522 | A/506 | 30 |
| 24 | 24 | A/627 | A/650 | A/503 | A/650 | A/639 | A/637 | 32 |
| 25 | 25 | A/556 | D/506 | A/634 | A/550 | D/532 | A/516 | - |
| 26 | 26 | A/594 | A/505 | A/603 | A/610 | A/600 | A/616 | 43 |
| 27 | 27 | C/658 | C/605 | C/674 | C/733 | C/722 | C/692 | 47 |
| 28 | 28 | C/719 | C/701 | A/630 | A/624 | A/620 | C/764 | 44 |
| 29 | 29 | C/641 | C/679 | C/637 | C/616 | C/643 | C/646 | 45 |
| 30 | 30 | C/654 | C/601 | C/629 | C/645 | C/731 | C/710 | 46 |
| 31 | 31 | C/734 | C/818 | C/612 | C/820 | C/782 | C/723 | 50 |
| 32 | 32 | C/726 | A/561 | C/606 | A/528 | C/764 | C/753 | 51 |
| 33 | 33 | A/521 | A/620 | A/548 | A/586 | A/560 | A/548 | 52 |
| 34 | 34 | C/669 | C/817 | C/806 | C/657 | C/693 | C/696 | 53 |
| 35 | 35 | A/606 | A/539 | A/524 | A/593 | A/585 | A/592 | 59 |
| 36 | 36 | D/579 | O/405 | D/625 | D/542 | D/566 | D/548 | 66 |
| 37 | 37 | G/267 | A/627 | A/600 | A/570 | - | A/577 | 119 |
| 38 | 38 | D/619 | D/562 | D/590 | D/622 | D/618 | D/623 | 117 |
| 39 | 39 | O/464 | O/409 | O/456 | O/510 | O/504 | O/339 | 122 |
| 40 | 40 | G/10 | O/439 | O/491 | O/493 | O/511 | O/384 | 121 |
| 41 | 41 | D/566 | D/543 | D/520 | O/463 | O/459 | D/558 | 67 |
| 42 | 42 | D/597 | D/516 | D/518 | D/587 | D/578 | D/608 | 68 |
| 43 | 43 | O/439 | O/438 | D/626 | O/468 | O/425 | D/579 | 69 |
| 44 | 44 | A/574 | A/590 | A/529 | A/557 | A/553 | A/554 | - |
| 45 | 45 | - | A/660 | - | A/501 | - | - | - |
| 46 | 46 | A/577 | C/697 | A/631 | A/648 | C/660 | A/623 | 54 |
| 47 | 47 | A/560 | A/617 | A/558 | A/553 | A/561 | A/521 | 55 |
| 48 | 48 | C/606 | C/630 | A/628 | C/660 | C/621 | C/631 | 56 |
| 49 | 49 | A/543 | C/619 | A/632 | A/537 | A/531 | A/504 | 57 |
| 50 | 50 | A/589 | A/580 | A/614 | A/535 | A/605 | A/590 | 58 |
| 51 | 51 | A/513 | A/548 | A/522 | A/601 | A/554 | A/531 | - |
| 52 | 52 | A/584 | A/555 | A/554 | A/563 | A/526 | A/553 | 31 |
| 53 | 53 | - | A/658 | A/571 | A/651 | - | A/620 | 39 |
| 54 | 54 | A/546 | A/538 | A/552 | A/598 | A/590 | A/608 | 33 |
| 55 | 55 | A/505 | V/313 | A/647 | A/556 | A/556 | A/524 | 34 |
| 56 | 56 | V/305 | V/320 | V/338 | V/319 | C/622 | C/669 | 35 |
| 57 | - | C/602 | V/369 | V/348 | C/643 | C/624 | - | - |
| 58 | - | V/344 | V/324 | C/803 | C/729 | V/346 | - | - |
| 59 | - | A/573 | A/512 | A/598 | A/558 | A/564 | D/575 | 36 |
| 60 | - | V/335 | U/323 | U/313 | V/353 | V/326 | U/207 | - |
| 61 | - | V/361 | U/358 | U/333 | U/309 | - | U/221 | 37 |
| 62 | - | G/203 | U/331 | U/328 | U/365 | U/248 | - | - |
| 63 | - | D/561 | D/613 | D/571 | D/534 | D/542 | D/528 | 190 |
| 64 | - | G/24 | B/750 | B/755 | B/903 | B/799 | B/863 | 102 |
| 65 | - | D/570 | D/503 | B/939 | D/572 | D/575 | D/573 | 101 |
| 66 | - | G/85 | U/367 | U/372 | U/307 | U/201 | - | - |
| 67 | - | A/593 | A/568 | A/575 | A/592 | A/601 | A/617 | 61 |
| 68 | - | D/513 | O/448 | O/518 | O/433 | D/519 | O/348 | 125 |
| 69 | - | O/422 | O/482 | O/503 | O/548 | O/483 | - | - |
| 70 | - | O/455 | O/472 | O/466 | O/470 | O/469 | O/363 | - |
| 71 | - | O/445 | O/516 | O/510 | O/416 | O/478 | O/357 | 154 |
| 72 | - | D/543 | D/593 | D/601 | D/571 | D/585 | A556 | 129 |
| 73 | - | O/428 | O/422 | O/422 | O/412 | O/446 | O/315 | - |

**Supplementary Table 2** | R_iso_ values among different datasets of the 1F (A) and 2F (B) data.

**A. 1F datasets**

|  | 1F (20 ns) | 1F (200 ns) | 1F (1 μs) | 1F (30 μs) | 1F (200 μs) | 1F (5 ms) |
| --- | --- | --- | --- | --- | --- | --- |
| Dark | 0.07 | 0.08 | 0.07 | 0.07 | 0.06 | 0.08 |
| 1F (20 ns) |  | 0.08 | 0.08 | 0.08 | 0.08 | 0.08 |
| 1F (200 ns) |  |  | 0.08 | 0.08 | 0.07 | 0.08 |
| 1F (1 μs) |  |  |  | 0.08 | 0.07 | 0.09 |
| 1F (30 μs) |  |  |  |  | 0.08 | 0.09 |
| 1F (200 μs) |  |  |  |  |  | 0.09 |

**B. 2F datasets**

|  | 2F (20 ns) | 2F (200 ns) | 2F (1 μs) | 2F (30 μs) | 2F (200 μs) | 2F (5 ms) |
| --- | --- | --- | --- | --- | --- | --- |
| 1F | 0.09 | 0.08 | 0.08 | 0.11 | 0.09 | 0.08 |
| 2F (20 ns) |  | 0.09 | 0.08 | 0.10 | 0.10 | 0.10 |
| 2F (200 ns) |  |  | 0.07 | 0.10 | 0.09 | 0.08 |
| 2F (1 μs) |  |  |  | 0.09 | 0.09 | 0.09 |
| 2F (30 μs) |  |  |  |  | 0.11 | 0.11 |
| 2F (200 μs) |  |  |  |  |  | 0.09 |

**Supplementary Table 3 |** **The intensities of the difference densities at various time points following 1F or 2F.**

| **Q_A_-Q_B_ area** | **1F** | | | | | | **2F** | | | | | |
| --- | --- | --- | --- | --- | --- | --- | --- | --- | --- | --- | --- | --- |
|  | **20 ns** | **200 ns** | **1 μs** | **30 μs** | **200 μs** | **5 ms** | **20 ns** | **200 ns** | **1 μs** | **30 μs** | **200 μs** | **5 ms** |
| Q_A_ head group (-) (A)* | -4.1 | -5.1 | -4.3 | -4.7 | -3.0 | -2.2 | -3.0 | -4.4 | -3.7 | -3.8 | -3.2 | -2.7 |
| Q_A_ head group (+) (A)* | 5.9 | 5.8 | 5.2 | 5.1 | 4.6 | 2.9 | 4.1 | 4.4 | 4.6 | 3.9 | 4.3 | 3.8 |
| Q_A_ head group (-) (B)* | -4.7 | -4.5 | -4.0 | -4.7 | -4.2 | -2.5 | -3.4 | -4.1 | -4.3 | -3.4 | -3.7 | -3.2 |
| Q_A_ head group (+) (B)* | 5.5 | 6.0 | 5.4 | 4.8 | 3.3 | 3.1 | 4.4 | 3.9 | 3.6 | 3.9 | 3.9 | 2.3 |
| Non-heme iron (-) (A) | -5.3 | -6.3 | -5.3 | -3.8 | -5.2 | -2.5 | -3.6 | -4.0 | -3.5 | -4.0 | -5.0 | 0.0 |
| Non-heme iron (+) (A) | 6.3 | 6.5 | 4.4 | 4.5 | 3.3 | 0.0 | 3.7 | 4.0 | 3.9 | 3.9 | 0.0 | 0.0 |
| Non-heme iron (-) (B) | -4.8 | -5.1 | -7.6 | -2.2 | -4.4 | -2.4 | -4.4 | -3.0 | -3.6 | -2.1 | -4.4 | -2.4 |
| Non-heme iron (+) (B) | 7.7 | 6.4 | 8.1 | 5.7 | 2.8 | 3.1 | 3.9 | 4.5 | 3.5 | 3.5 | 4.4 | 0.0 |
| BCT (-) (A) | 0.0 | -4.6 | -4.9 | -2.7 | -4.1 | -2.3 | 0.0 | 0.0 | 0.0 | 0.0 | 0.0 | 0.0 |
| BCT (+) (A) | 2.8 | 2.9 | 0.0 | 5.1 | 3.8 | 5.3 | 3.0 | 0.0 | 0.0 | 2.7 | 0.0 | 0.0 |
| BCT (-) (A) | -4.1 | 2.2 | -3.1 | 2.6 | 4.6 | 4.6 | 3.3 | 2.4 | 0.0 | 0.0 | 0.0 | 0.0 |
| BCT (+) (B) | 4.0 | 4.0 | 2.8 | -3.4 | -3.1 | -3.0 | -3.6 | 0.0 | 0.0 | 0.0 | 0.0 | 0.0 |
| Q_B_ head group (A) | 0.0 | 3.1 | 3.4 | 3.4 | 3.7 | 5.0 | 3.2 | 3.2 | 0.0 | 2.2 | 2.9 | 5.0 |
| Q_B_ head group (B) | 2.6 | 3.2 | 2.6 | 3.4 | 3.8 | 4.4 | 3.5 | 0.0 | 0.0 | 0.0 | 3.1 | 3.2 |
| **Y_Z_ area** | **1F** | | | | | | **2F** | | | | | |
|  | **20 ns** | **200 ns** | **1 μs** | **30 μs** | **200 μs** | **5 ms** | **20 ns** | **200 ns** | **1 μs** | **30 μs** | **200 μs** | **5 ms** |
| PD1-Mg (A) | 0.0 | 4.9 | 0.0 | 0.0 | 0.0 | 0.0 | 0.0 | 0.0 | 0.0 | 0.0 | 0.0 | 0.0 |
| PD1-Mg (B) | 0.0 | 4.0 | 0.0 | 0.0 | 0.0 | 0.0 | 0.0 | 0.0 | 0.0 | 0.0 | 0.0 | 0.0 |
| D1-H190 (-) (A) | -3.5 | -5.2 | -4.7 | -3.7 | -2.8 | 0.0 | -2.3 | -2.9 | -3.7 | -4.3 | -5.3 | -2.9 |
| D1-H190 (+) (A) | 2.9 | 6.4 | 5.3 | 4.1 | 0.0 | 0.0 | 2.3 | 3.0 | 3.7 | 3.5 | 3.4 | 2.9 |
| D1-H190 (-) (B) | -2.6 | -5.5 | -3.4 | -3.5 | -2.2 | 0.0 | -2.2 | -2.5 | -5.0 | -2.7 | -3.7 | -3.6 |
| D1-H190 (+) (B) | 2.8 | 6.0 | 4.2 | 3.9 | 2.0 | 3.4 | 3.2 | 2.8 | 2.6 | 3.7 | 4.6 | 2.5 |
| D1-F186 (-) (A) | -3.4 | -4.7 | -6.6 | -3.6 | -2.7 | -1.9 | -2.4 | -2.9 | -3.1 | -3.6 | -3.8 | 0.0 |
| D1-F186 (+) (A) | 3.6 | 3.9 | 4.8 | 5.3 | 2.3 | 2.7 | 0.0 | 3.2 | 3.6 | 3.8 | 3.3 | 2.2 |
| D1-F186 (-) (B) | -3.9 | -4.1 | -4.3 | -3.7 | -4.3 | -2.1 | -3.8 | -2.9 | -3.3 | -3.0 | -3.7 | -4.9 |
| D1-F186 (+) (B) | 4.0 | 4.4 | 5.4 | 5.2 | 3.6 | 2.9 | 4.1 | 3.3 | 3.0 | 6.1 | 4.4 | 3.7 |
| Yz (-) (A) | -4.1 | -7.1 | -7.1 | -6.0 | -3.6 | -2.8 | -2.8 | -4.5 | -6.1 | -5.2 | -4.4 | -3.3 |
| Yz (+) (A) | 3.3 | 6.2 | 4.7 | 4.4 | 3.2 | 2.7 | 3.2 | 4.2 | 5.4 | 4.6 | 4.1 | 2.9 |
| Yz (-) (B) | -4.6 | -7.2 | -7.0 | -5.7 | -3.5 | -2.8 | -4.1 | -4.2 | -4.5 | -3.4 | -3.0 | -2.5 |
| Yz (+) (B) | 4.1 | 4.8 | 5.4 | 4.9 | 3.7 | 2.9 | 3.2 | 3.8 | 4.4 | 3.8 | 3.6 | 3.3 |
| D1-Q165 (-) (A) | -4.7 | -6.8 | -6.4 | -5.2 | -3.7 | -2.3 | -3.0 | -5.3 | -7.1 | -5.9 | -4.0 | -2.3 |
| D1-Q165 (+) (A) | 3.1 | 5.3 | 4.2 | 6.1 | 2.4 | 3.4 | 3.5 | 4.3 | 5.3 | 5.8 | 4.4 | 2.2 |
| D1-Q165 (-) (B) | -2.7 | -4.1 | -4.2 | -5.5 | -3.9 | 0.0 | -2.3 | -3.3 | -5.8 | -4.9 | -3.9 | 0.0 |
| D1-Q165 (+) (B) | 4.3 | 3.8 | 5.5 | 5.3 | 4.0 | 2.7 | 3.6 | 4.0 | 6.0 | 6.4 | 3.6 | 0.0 |
| W7 (A) | -2.4 | -3.7 | -8.2 | -7.2 | -2.6 | 0.0 | 0.0 | -2.9 | -3.7 | -5.7 | -4.7 | -2.2 |
| W7 (B) | 0.0 | -4.9 | -7.3 | -5.2 | -3.2 | 0.0 | 0.0 | -3.0 | -2.9 | -3.7 | -5.8 | -3.5 |
| W3 (A) | 2.7 | 4.7 | 2.6 | 0.0 | 0.0 | 0.0 | 0.0 | 0.0 | 0.0 | -3.9 | -4.2 | 0.0 |
| W3 (B) | 0.0 | 0.0 | 0.0 | -3.5 | 0.0 | 0.0 | 0.0 | 0.0 | 0.0 | -2.5 | -4.8 | 0.0 |
| W4 (A) | 0.0 | 0.0 | 0.0 | -3.1 | -2.9 | -2.8 | 0.0 | 0.0 | 0.0 | -4.5 | -5.6 | 0.0 |
| W4 (B) | 0.0 | 0.0 | 0.0 | 0.0 | -3.5 | -3.7 | 0.0 | -2.3 | -2.2 | -2.9 | -6.1 | 0.0 |
| **OEC** | **1F** | | | | | | **2F** | | | | | |
|  | **20 ns** | **200 ns** | **1 μs** | **30 μs** | **200 μs** | **5 ms** | **20 ns** | **200 ns** | **1 μs** | **30 μs** | **200 μs** | **5 ms** |
| Mn1 (-) (A) | 0.0 | 0.0 | 0.0 | 0.0 | 0.0 | 0.0 | -4.7 | -3.2 | -4.9 | -6.5 | -8.5 | -4.0 |
| Mn1 (+) (A) | 3.7 | 8.8 | 2.8 | 0.0 | 3.2 | 6.8 | 3.2 | 3.0 | 4.6 | 6.3 | 8.0 | 9.0 |
| Mn1 (-) (B) | -4.8 | -2.7 | -2.7 | 0.0 | 0.0 | 0.0 | -2.1 | -2.8 | -3.9 | -4.5 | -7.3 | -6.6 |
| Mn1 (+) (B) | 5.8 | 5.0 | 0.0 | 0.0 | 3.9 | 4.2 | 3.6 | 2.1 | 3.5 | 3.9 | 8.3 | 8.8 |
| Mn4 (-) (A) | -3.9 | -4.2 | -2.9 | 0.0 | -6.0 | -3.6 | 0.0 | 0.0 | 0.0 | -4.3 | -6.7 | -8.2 |
| Mn4 (+) (A) | 5.7 | 4.9 | 4.4 | 4.4 | 12.3 | 9.9 | 3.0 | 0.0 | 0.0 | 4.6 | 5.9 | 8.2 |
| Mn4 (-) (B) | -3.1 | -3.7 | 0.0 | 0.0 | -6.3 | -5.7 | -2.9 | -3.0 | 0.0 | -4.5 | -8.2 | -7.6 |
| Mn4 (+) (B) | 5.0 | 4.9 | 0.0 | 6.0 | 10.7 | 9.9 | 4.2 | 3.3 | 3.0 | 4.8 | 5.2 | 4.4 |
| Ca (-) (A) | 0.0 | 0.0 | 0.0 | -4.9 | -3.6 | -6.0 | -3.3 | -3.6 | -4.1 | -7.3 | -8.6 | -4.7 |
| Ca (+) (A) | 2.8 | 5.5 | 2.4 | 5.2 | 8.4 | 6.2 | 2.4 | 0.0 | 0.0 | 0.0 | 0.0 | 0.0 |
| Ca (-) (B) | 0.0 | 0.0 | 0.0 | -5.6 | -6.8 | -4.9 | 0.0 | 0.0 | -3.3 | -5.2 | -6.7 | 0.0 |
| Ca (+) (B) | 3.9 | 4.6 | 4.7 | 4.3 | 6.9 | 5.3 | 0.0 | 0.0 | 0.0 | 0.0 | 0.0 | 0.0 |
| O6*(A) | 0.0 | 0.0 | 0.0 | 0.0 | 0.0 | 0.0 | 0.0 | 3.3 | 6.0 | 6.0 | 6.0 | 2.8 |
| O6*(B) | 0.0 | 0.0 | 0.0 | 0.0 | 0.0 | 0.0 | 0.0 | 2.6 | 3.7 | 4.5 | 3.3 | 0.0 |
| O6 (A) | 0.0 | 0.0 | 0.0 | 0.0 | 0.0 | 0.0 | 2.3 | 2.2 | 3.0 | 3.7 | 7.8 | 9.0 |
| O6 (B) | 0.0 | 0.0 | 0.0 | 0.0 | 0.0 | 0.0 | 0.0 | 3.0 | 2.0 | 3.5 | 5.4 | 5.4 |
| **O1-channel** | **1F** | | | | | | **2F** | | | | | |
|  | **20 ns** | **200 ns** | **1 μs** | **30 μs** | **200 μs** | **5 ms** | **20 ns** | **200 ns** | **1 μs** | **30 μs** | **200 μs** | **5 ms** |
| D1-D342 (-) (A) | -4.0 | -6.0 | -5.1 | -5.2 | -6.6 | -4.0 | 0.0 | -3.1 | 0.0 | 0.0 | -2.2 | -2.8 |
| D1-D342 (+) (A) | 4.3 | 6.6 | 5.5 | 6.2 | 7.1 | 5.6 | 2.9 | 3.7 | 0.0 | 0.0 | 3.0 | 3.9 |
| D1-D342 (-) (B) | -5.1 | -4.1 | -6.5 | -5.0 | -6.9 | -5.4 | -2.1 | 0.0 | 0.0 | 0.0 | 0.0 | 0.0 |
| D1-D342 (+) (B) | 6.7 | 7.3 | 7.5 | 7.6 | 7.5 | 7.1 | 2.6 | 2.4 | 2.6 | 2.9 | 0.0 | 0.0 |
| D1-E329 (-) (A) | -3.6 | -4.9 | -6.4 | -4.0 | -2.4 | -3.3 | -3.5 | -2.0 | -3.0 | -3.7 | -2.4 | 3.8 |
| D1-E329 (+) (A) | 2.9 | 4.9 | 6.0 | 4.5 | 2.5 | 2.7 | 2.5 | 3.9 | 4.7 | 3.8 | 4.5 | -3.0 |
| D1-E329 (-) (B) | -2.4 | -2.8 | -6.0 | -2.5 | 0.0 | 0.0 | 0.0 | -2.0 | -3.2 | 0.0 | -2.3 | 3.7 |
| D1-E329 (+) (B) | 0.0 | 2.5 | 4.8 | 3.6 | 2.6 | 0.0 | 0.0 | 0.0 | 2.0 | 2.4 | 0.0 | -3.0 |
| D1-E189-main chain (-) (A) | 0.0 | -3.1 | -2.7 | -2.7 | 0.0 | 0.0 | 0.0 | -2.6 | -3.8 | -3.8 | -4.9 | -6.8 |
| D1-E189-main chain(+) (A) | 2.2 | 2.9 | 2.5 | 2.9 | 2.2 | 2.1 | 0.0 | 0.0 | 0.0 | 3.3 | 4.1 | 4.7 |
| D1-E189-main chain(-) (B) | 0.0 | -2.7 | 0.0 | 0.0 | 0.0 | 0.0 | 0.0 | -2.7 | 0.0 | -2.4 | -4.1 | -4.4 |
| D1-E189-main chain(+) (B) | 0.0 | 4.2 | 2.1 | 3.9 | 2.1 | 0.0 | 2.1 | 2.7 | 3.7 | 0.0 | 3.9 | 5.5 |
| D1-V410 (-) (A) | -3.5 | -3.8 | -4.0 | -3.1 | -3.2 | -2.6 | 0.0 | -2.3 | -2.8 | -3.5 | -3.9 | -5.1 |
| D1-V410 (+) (A) | 2.3 | 0.0 | 0.0 | 0.0 | 0.0 | 0.0 | 0.0 | 0.0 | 0.0 | 2.7 | 3.9 | 4.6 |
| D1-V410 (-) (B) | -3.5 | -3.8 | -4.1 | -3.2 | -3.2 | -2.7 | -2.1 | 0.0 | -2.3 | 0.0 | -3.1 | -3.7 |
| D1-V410 (+) (B) | 2.3 | 0.0 | 0.0 | 0.0 | 0.0 | 0.0 | 0.0 | 0.0 | 0.0 | 3.4 | 3.6 | 4.8 |
| PsbU-K104 (-) (A) | -2.8 | -4.6 | -6.6 | -6.5 | -4.8 | -3.8 | 0.0 | -2.9 | -3.3 | -4.3 | -2.6 | 0.0 |
| PsbU-K104 (+) (A) | 3.1 | 3.4 | 4.3 | 4.1 | 2.9 | 0.0 | 0.0 | 0.0 | 0.0 | 0.0 | 0.0 | 0.0 |
| PsbU-K104 (-) (B) | 0.0 | -3.7 | -5.9 | -5.1 | -3.5 | -2.2 | -2.5 | -2.2 | -4.2 | -2.1 | -2.9 | 0.0 |
| PsbU-K104 (+) (B) | 0.0 | 0.0 | 2.6 | 2.3 | 3.3 | 0.0 | 0.0 | 2.4 | 2.8 | 0.0 | 0.0 | 0.0 |
| W10 (A) | 3.1 | 3.9 | 3.0 | -6.5 | -10.5 | -8.2 | -4.2 | -4.1 | 0.0 | 0.0 | 0.0 | 0.0 |
| W10 (B) | 0.0 | 4.8 | 0.0 | -4.6 | -7.4 | -6.8 | 0.0 | 0.0 | 0.0 | 0.0 | -2.0 | 0.0 |
| W20 (A) | -4.4 | -6.4 | -6.5 | -6.8 | -6.9 | -3.9 | 0.0 | 0.0 | -3.1 | 0.0 | -5.1 | -4.2 |
| W20 (B) | -4.6 | -5.5 | -6.3 | -6.2 | -6.8 | -3.8 | 0.0 | 0.0 | 0.0 | -2.4 | -3.2 | -3.5 |
| W21 (-) (A) | 0.0 | -2.4 | -3.0 | -3.7 | -3.1 | -3.9 | 0.0 | 0.0 | 0.0 | 0.0 | 0.0 | 0.0 |
| W21 (+) (A) | 3.0 | 6.1 | 5.2 | 4.1 | 3.2 | 2.7 | 0.0 | 0.0 | 0.0 | 0.0 | 0.0 | -3.3 |
| W21 (-) (B) | 0.0 | 0.0 | -2.3 | -2.8 | -3.0 | 0.0 | 0.0 | 0.0 | -2.7 | -2.5 | -2.7 | 0.0 |
| W21 (+) (B) | 2.2 | 2.8 | 4.3 | 0.0 | 0.0 | 0.0 | 0.0 | 0.0 | 0.0 | 0.0 | 0.0 | 0.0 |
| W23 (A) | 4.7 | 4.2 | 3.5 | 3.7 | 0.0 | 0.0 | 0.0 | 0.0 | -2.1 | -2.6 | -3.5 | 0.0 |
| W23 (B) | 2.2 | 2.8 | 4.3 | -2.8 | -3.0 | 0.0 | 0.0 | 0.0 | -2.8 | -2.5 | -2.7 | -2.1 |
| W24 (A) | 2.5 | 1.3 | -2.2 | -2.6 | -2.4 | -3.9 | 0.0 | 0.0 | 0.0 | 0.0 | -4.8 | -4.1 |
| W24 (B) | 0.0 | 0.0 | -2.4 | -4.4 | -2.4 | 0.0 | 0.0 | 0.0 | 0.0 | -2.1 | -4.7 | -2.4 |
| W25 (A) | 0.0 | -2.8 | -5.3 | -4.5 | -2.7 | -3.7 | 0.0 | -2.1 | -2.2 | -2.0 | -3.7 | -4.4 |
| W25 (B) | -2.1 | -2.7 | -3.1 | -2.7 | 0.0 | 0.0 | 0.0 | 0.0 | 0.0 | -2.4 | -3.2 | -2.9 |
| W26 (A) | -3.2 | -5.8 | -6.1 | -5.9 | -4.4 | -4.2 | 0.0 | 0.0 | -3.7 | -3.4 | -3.8 | -3.7 |
| W26 (B) | -4.3 | -4.3 | -6.4 | -4.9 | -3.9 | -3.4 | 0.0 | -3.0 | -3.3 | -3.5 | -3.4 | 0.0 |
| W28 (A) | 2.3 | 2.9 | -2.5 | 0.0 | 0.0 | 0.0 | 0.0 | 0.0 | 0.0 | -4.1 | -5.0 | 0.0 |
| W28 (B) | 2.3 | 0.0 | 0.0 | -2.4 | 0.0 | -2.6 | -2.4 | 0.0 | 0.0 | 0 | -3.8 | 0.0 |
| W39 (A) | 2.5 | 4.6 | 4.2 | -3.3 | 4.8 | 0.0 | 0.0 | 0.0 | -2.5 | -3.8 | -4.3 | -2.5 |
| W39 (B) | 2.1 | 3.2 | 0.0 | -2.5 | 0.0 | 2.8 | 0.0 | 0.0 | 0.0 | -3.1 | -3.2 | -3.0 |
| W40' (A) | 0.0 | -4.2 | -3.9 | -4.2 | -6.0 | 0.0 | 0.0 | 0.0 | 0.0 | 0.0 | -2.6 | 0.0 |
| W40' (B) | 0.0 | -2.3 | -3.1 | -4.6 | -4.9 | -3.2 | 0.0 | 0.0 | 0.0 | -2.6 | -2.5 | 0.0 |
| **Cl-1-channel** | **1F** | | | | | | **2F** | | | | | |
|  | **20 ns** | **200 ns** | **1 μs** | **30 μs** | **200 μs** | **5 ms** | **20 ns** | **200 ns** | **1 μs** | **30 μs** | **200 μs** | **5 ms** |
| Cl-1 (-) (A) | -3.5 | -3.6 | -2.4 | -4.8 | -4.7 | -3.0 | -3.3 | -2.9 | -2.4 | -5.1 | -6.8 | -3.4 |
| Cl-1 (+) (A) | 5.0 | 7.2 | 4.3 | 4.1 | 5.9 | 4.3 | 2.2 | 2.8 | 2.9 | 3.0 | 4.8 | 3.9 |
| Cl-1 (-) (B) | -2.7 | -4.6 | -3.1 | -4.0 | -4.5 | -2.6 | -2.6 | 0.0 | 0.0 | -3.5 | -5.5 | -3.8 |
| Cl-1 (+) (B) | 3.4 | 4.7 | 3.8 | 4.0 | 5.3 | 4.1 | 3.5 | 3.0 | 3.6 | 3.1 | 3.0 | 3.0 |
| D1-N181 (-) (A) | -3.1 | -3.8 | -3.0 | -3.6 | -3.1 | -2.0 | 0.0 | 0.0 | -3.0 | 0.0 | -2.4 | 0.0 |
| D1-N181 (+) (A) | 2.6 | 2.7 | 3.8 | 3.9 | 2.6 | 0.0 | 0.0 | 3.3 | 3.1 | 4.4 | 5.7 | 2.7 |
| D1-N181 (-) (B) | -2.5 | -3.4 | -3.1 | -3.7 | -3.2 | -2.8 | -3.4 | 0.0 | -2.2 | -2.5 | -2.4 | -3.4 |
| D1-N181 (+) (B) | 0.0 | 2.0 | 0.0 | 2.7 | 2.5 | 0.0 | 0.0 | 0.0 | 0.0 | 0.0 | 3.4 | 2.6 |
|  |  |  |  |  |  |  |  |  |  |  |  |  |
| **O4-channel** |  |  |  |  |  |  |  |  |  |  |  |  |
| W16 (A) | -4.8 | -4.6 | -7.6 | -12.5 | -17.0 | -14.3 | 2.8 | 4.2 | 2.9 | 3.5 | 4.0 | 4.8 |
| W16 (B) | -4.0 | -5.7 | -5.8 | -11.5 | -15.8 | -12.3 | 2.1 | 0.0 | 0.0 | 0.0 | 0.0 | 2.3 |

The listed values are provided in units of σ (= root mean square of electron density). Any values below 2 σ were counted as zero. The color scheme used is green for positive difference densities and orange for negative difference densities. The intensity of the color corresponds to the strength of the difference density, with darker shades indicating higher values (counted separately for each designated area). (A) and (B) refer to A monomer and B monomer, respectively.


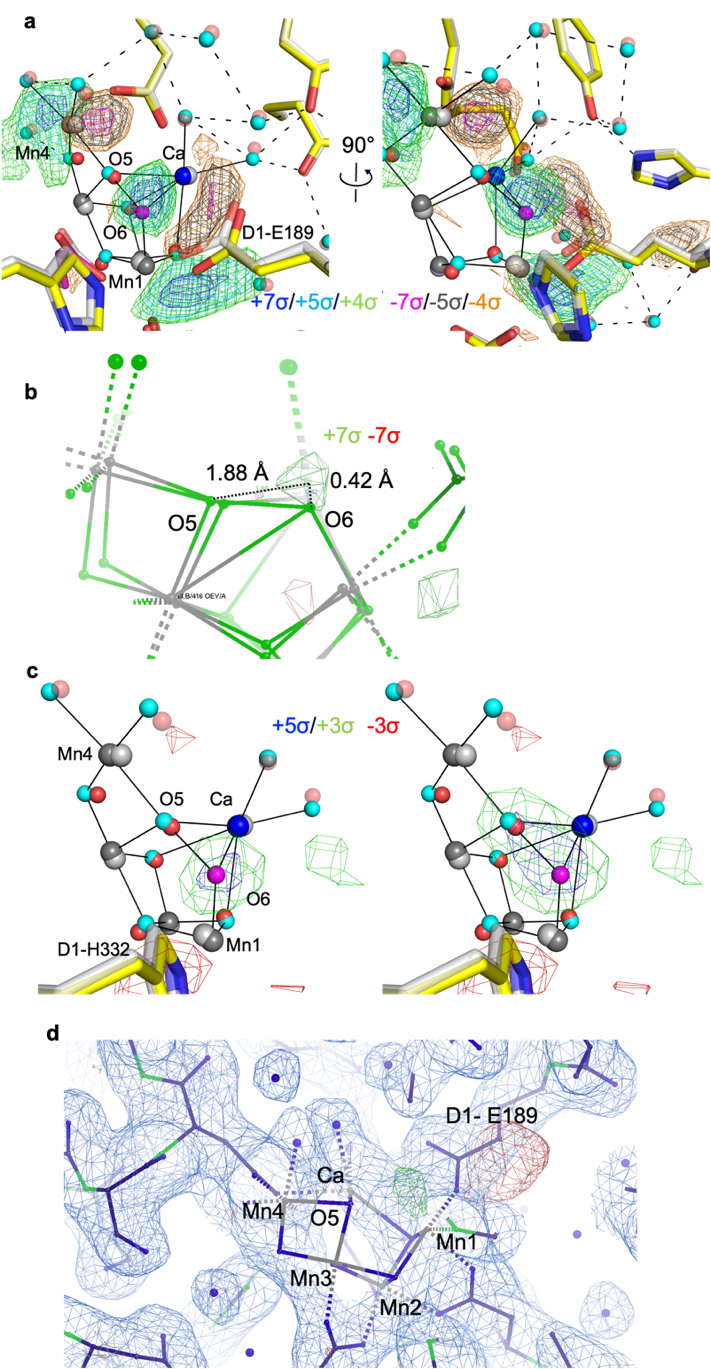


**Supplementary Fig. 1 | Investigation of O6 insertion into OEC at a delay time of 5 ms after the second flash.**

**(a)** and **(b)** shows the model of 2F (5 ms) superposed with the *F*_o_^2F(5ms)^-*F*_o_^1F^ difference *Fourier* maps. Map code and contour level are indicated in the figures. **(c)** displays Polder omit maps, with O6 omitted (left panel) and both O6 and the B conformation of O5 omitted (right panel). **(d)** Refinement of the OEC consisting of single O5 against the 2F data. 2Fo-Fc map contoured at 1.0 σ is shown in blue mesh and *F*_o_-*F*_c_ map contoured at ±3.0 σ is shown in green and red mesh. The residual densities near O5 and Mn1 are colored green and have a peak height of 3.45 σ, indicative of insertion of a new oxygen atom (O6). The Mn4-O5 distance was refined to be 2.48 Å.
