# Supplementary material for: Oxygen-evolving photosystem II structures during S1–S2–S3 transitions
Source: Nature. 2024 Jan 31;626(7999):670–7. doi: 10.1038/s41586-023-06987-5 (PMC10866707; doi:10.1038/s41586-023-06987-5)
Supplement: Supplementary file 2 — Reporting Summary [file 41586_2023_6987_MOESM2_ESM.pdf]

nature portfolio

Corresponding author(s): Jian-Ren Shen, Michihiro Suga

Last updated by author(s): Nov 23, 2023

Reporting Summary

Nature Portfolio wishes to improve the reproducibility of the work that we publish. This form provides structure for consistency and transparency in reporting. For further information on Nature Portfolio policies, see our Editorial Policies and the Editorial Policy Checklist.

Statistics

For all statistical analyses, confirm that the following items are present in the figure legend, table legend, main text, or Methods section.

n/a	Confirmed	
☐	☒	The exact sample size (*n*) for each experimental group/condition, given as a discrete number and unit of measurement
☒	☐	A statement on whether measurements were taken from distinct samples or whether the same sample was measured repeatedly
☒	☐	The statistical test(s) used AND whether they are one- or two-sided<br>*Only common tests should be described solely by name; describe more complex techniques in the Methods section.*
☒	☐	A description of all covariates tested
☒	☐	A description of any assumptions or corrections, such as tests of normality and adjustment for multiple comparisons
☐	☒	A full description of the statistical parameters including central tendency (e.g. means) or other basic estimates (e.g. regression coefficient) AND variation (e.g. standard deviation) or associated estimates of uncertainty (e.g. confidence intervals)
☒	☐	For null hypothesis testing, the test statistic (e.g. *F*, *t*, *r*) with confidence intervals, effect sizes, degrees of freedom and *P* value noted<br>*Give P values as exact values whenever suitable.*
☒	☐	For Bayesian analysis, information on the choice of priors and Markov chain Monte Carlo settings
☒	☐	For hierarchical and complex designs, identification of the appropriate level for tests and full reporting of outcomes
☒	☐	Estimates of effect sizes (e.g. Cohen's *d*, Pearson's *r*), indicating how they were calculated

Our web collection on statistics for biologists contains articles on many of the points above.

Software and code

Policy information about availability of computer code

Data collection	Cheetah (https://github.com/keitaroyam/cheetah), CrystFEL (version: 0.6.3)
Data analysis	CrystFEL (version: 0.6.3), dials.still_process in DIALs (version: 1.16), Publicly available CCP4 modules (CCP4 7.1.015) including CCTBX, SFTOOLS, Truncate, CAD, SCALEIT, and FFT. Packages in phenix (version: 1.19.2-4158) including cxi.merge and Phaser (2.8.3). Coot (version: 0.9.8.8), Pymol (version: 2.5.4)

For manuscripts utilizing custom algorithms or software that are central to the research but not yet described in published literature, software must be made available to editors and reviewers. We strongly encourage code deposition in a community repository (e.g. GitHub). See the Nature Portfolio guidelines for submitting code & software for further information.

Data

Policy information about availability of data

All manuscripts must include a data availability statement. This statement should provide the following information, where applicable:

- Accession codes, unique identifiers, or web links for publicly available datasets
- A description of any restrictions on data availability
- For clinical datasets or third party data, please ensure that the statement adheres to our policy

The atomic coordinates and structure factors have been deposited in the Protein Data Bank under the following IDs: 8IR5 for 0F (dark, ground state for the Δt1 structures), 8IR6 for Δt1 = 20 ns, 8IR7 for Δt1 = 200 ns, 8IR8 for Δt1 = 1 µs, 8IR9 for Δt1 = 30 µs, 8IRA for Δt1 = 200 µs, 8IRB for Δt1 = 5 ms, 8IRC for 1F (ground state

for the Δt2 structures), 8IRD for Δt2 = 20 ns, 8IRE for Δt2 = 200 ns, 8IRF for Δt2 = 1 µs, 8IRG for Δt2 = 30 µs, 8IRH for Δt2 = 200 µs, and 8IRI for Δt2 = 5 ms. All other data with a PDB code used in this study are adopted from the PDB databank.

Research involving human participants, their data, or biological material

Policy information about studies with human participants or human data. See also policy information about sex, gender (identity/presentation), and sexual orientation and race, ethnicity and racism.

Reporting on sex and gender	n/a
Reporting on race, ethnicity, or other socially relevant groupings	n/a
Population characteristics	n/a
Recruitment	n/a
Ethics oversight	n/a

Note that full information on the approval of the study protocol must also be provided in the manuscript.

Field-specific reporting

Please select the one below that is the best fit for your research. If you are not sure, read the appropriate sections before making your selection.

☒ Life sciences ☐ Behavioural & social sciences ☐ Ecological, evolutionary & environmental sciences

For a reference copy of the document with all sections, see nature.com/documents/nr-reporting-summary-flat.pdf

Life sciences study design

All studies must disclose on these points even when the disclosure is negative.

Sample size	The sample size is determined based on the number of diffraction images that produced reasonably high resolution and multiplicity or number of unique diffractions in each time-resolved serial crystallography data set. The microcrystals obtained were reproducible, and the diffraction data was processed with standard crystallographic software (Methods section), which resulted in standard data statistics as shown in Extended Data Table 1.
Data exclusions	No data was excluded; for details of the data analysis statistics, see Extended Data Table 1.
Replication	Thousands of microcrystals were used to obtain a full diffraction dataset, which showed that the results are well reproduced. The repetition rate of a specific dataset was shown in Extended Data Table 1, in which, it was shown that the repetition rate is over 100 even for the highest resolution shell for each of the dataset.
Randomization	The microcrystals used for the diffraction experiments were not chosen, and diffraction data from good microcrystals were used for the structural analysis. The criterion for choosing good diffraction data is based on the resolution as well as their deviations from standard cell parameters, as described in the Methods section.
Blinding	Not applicable. All experimental maps were included in the statistical analysis.

Reporting for specific materials, systems and methods

We require information from authors about some types of materials, experimental systems and methods used in many studies. Here, indicate whether each material, system or method listed is relevant to your study. If you are not sure if a list item applies to your research, read the appropriate section before selecting a response.

Materials & experimental systems

n/a	Involved in the study
☒	☐ Antibodies
☒	☐ Eukaryotic cell lines
☒	☐ Palaeontology and archaeology
☒	☐ Animals and other organisms
☒	☐ Clinical data
☒	☐ Dual use research of concern
☒	☐ Plants

Methods

n/a	Involved in the study
☒	☐ ChIP-seq
☒	☐ Flow cytometry
☒	☐ MRI-based neuroimaging

Plants

Seed stocks	n/a
Novel plant genotypes	n/a
Authentication	n/a
